# Multi-crosslinked photocurable hydrogel for dry eye therapy: dual ROS scavenging and macrophage reprogramming for mitochondrial redox homeostasis and inflammation regulation through HIF-1/NF-κB suppression

**DOI:** 10.1016/j.ajps.2026.101209

**Published:** 2026-10-18

**Authors:** Qinghe Zhang, Ke Yan, Panqin Ma, Weilu He, Minyan Wei, Qiuping Liu, Yi Han, Zuguo Liu

**Affiliations:** a Department of Ophthalmology, The First Affiliated Hospital of University of South China, Hengyang Medical School, University of South China, Hengyang 421001, China; b Xiamen University Affiliated Xiamen Eye Center, Fujian Provincial Key Laboratory of Ophthalmology and Visual Science, Fujian Engineering and Research Center of Eye Regenerative Medicine, Eye Institute of Xiamen University, School of Medicine, Xiamen University, Xiamen 361005, China

**Keywords:** Photocurable hydrogels, Multi-crosslink, Dry eye, Inflammation, Mitochondrial redox homeostasis, Oxidative stress

## Abstract

As air pollution intensifies and the use of electronic devices proliferates, the incidence of dry eye (DE) has surged, posing a significant burden on visual health. Given its complex pathogenesis and multifaceted etiology, achieving optimal therapeutic outcomes through a single treatment approach remains elusive. Hydrogel-based drug delivery systems have garnered considerable research attention. Here, a photocurable hydrogel (CsA@F127DA/DA-PEG-GelMA), that possesses both anti-inflammatory and antioxidant properties and utilizes advanced multi-crosslinking technology, was developed. This novel formulation significantly enhances the delivery efficiency of the hydrophobic immunosuppressant cyclosporine A (CsA). Furthermore, the incorporation of dopamine (DA) not only provides potent antioxidant and, ROS-scavenging capabilities, addressing ocular surface inflammatory disorders, but also endows the multifunctional hydrogel system with exceptional adhesion properties. The results showed that CsA@F127DA/DA-PEG-GelMA could not only inhibit the activation of NF-κB signaling pathway, regulate the polarization of macrophages and inhibit conjunctival inflammatory infiltration, but also inhibited the activation of HIF-1α signaling pathway, regulated glucose metabolism reprogramming, reduced the production of ROS, and thus maintained mitochondrial redox homeostasis. In summary, the dual inhibitory effect of CsA@F127DA/DA-PEG-GelMA on inflammation and oxidative stress effectively ameliorated the vicious cycle of DE, increased tear secretion, and significantly alleviated DE symptoms.

## Graphical abstract

**Figure:**
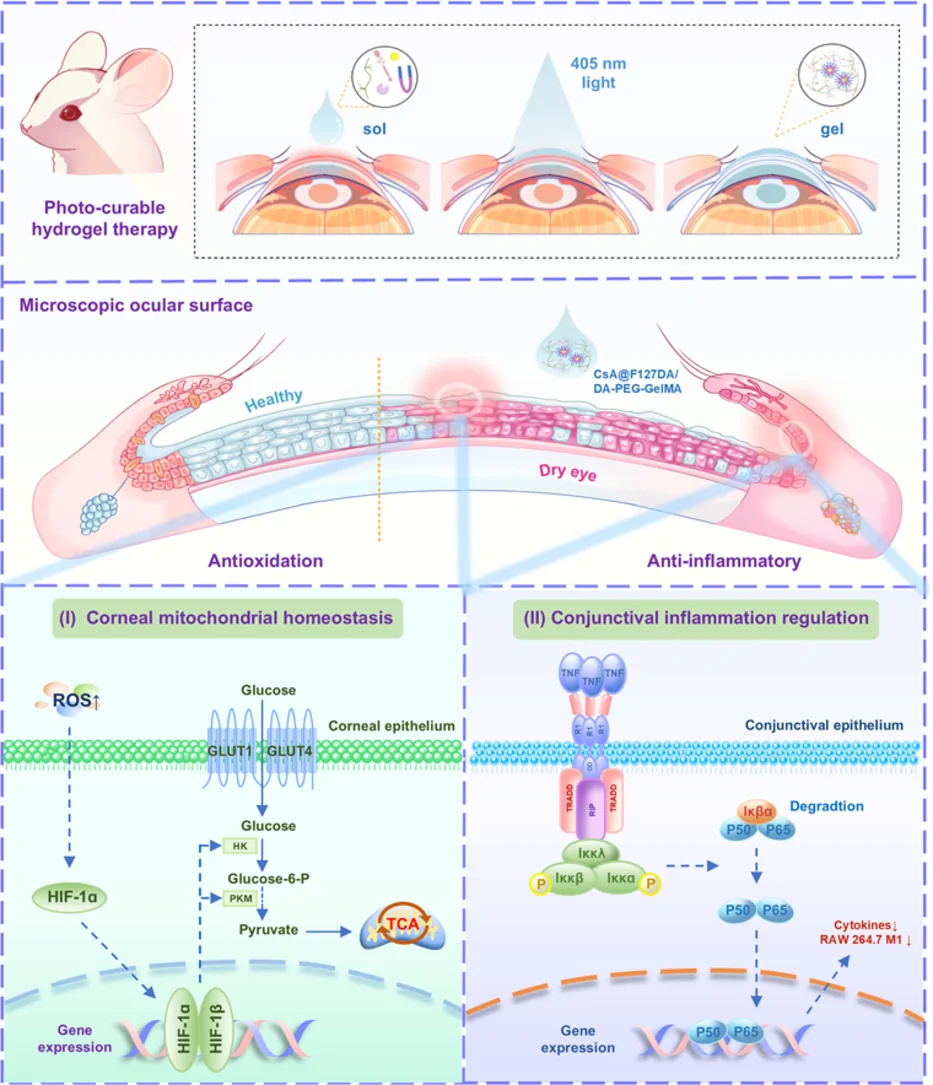

## 1 Introduction

Dry eye (DE) is a multifactorial disease of the ocular surface with a wide range of symptoms [1]. Amidst the deteriorating environment and escalating life pressures, the incidence of DE is rising annually, which incurs substantial economic burdens [2]. Inflammation, as an early innate immune response and subsequent adaptive response process, has been identified as a key factor in the development of DE. Macrophages also play an important role in inducing and regulating the inflammatory microenvironment of DE [3]. Cyclosporine A (CsA), a calcineurin inhibitor, has potent immunosuppressive effects. It also has a certain regulatory effect on macrophages [[4], [5], [6]]. However, CsA, being a highly hydrophobic cyclic peptide, poses a significant challenge in clinical applications [7]. Recent studies have shown that oxidative stress is also pivotal in exacerbating the DE vicious cycle. The resulting imbalance in mitochondrial homeostasis further contributes to the pathology of DE, leading to the imbalance of the ocular surface microenvironment [8,9]. Healthy mitochondria play an important role in maintaining the redox homeostasis of the ocular surface [10,11]. Therefore, a dual-approach strategy of mitigating inflammatory infiltration with immunosuppressive agents and maintaining mitochondrial redox homeostasis by scavenging excess reactive oxygen species (ROS) through antioxidants, plays a pivotal role in addressing DE [12].

Current medications for DE primarily consist of eye drops that directly interface with the ocular surface. However, corneal epithelial tight junctions, tears and mechanical actions including blinking result in the low absorption and utilization of eye drops [13]. Recently, drug delivery systems such as micelles and hydrogels have been developed to improve drug solubility and retention time [14]. The photocurable hydrogel-based drug delivery system is one of these systems of great interest. It is capable of undergoing a phase transition from a liquid solution to a solid hydrogel state upon exposure to UV or visible light irradiation [15]. Utilizing light to achieve high-efficiency loading of drugs into the hydrogel network ensures effective delivery, facilitates long-lasting slow release of ocular drugs, and enhances the bioavailability of the medications. F127DA is formed by the introduction of a acrylate groups into the molecular chain of F127, which has the ability to self-assemble into nano-micelles in water, and has been widely used to enhance the solubility of hydrophobic drugs to achieve hydrophobic drug-loaded functionality [7,16]. GelMA is crafted by grafting methacrylic anhydride (MA) onto gelatin via side-chain amino, resulting in a material that possesses exceptional biocompatibility and highly adjustable physicochemical properties. It is extensively utilized in the realm of drug delivery and has found applications across a diverse spectrum, including wound dressings and bioengineering [17,18].

The current study aims to develop a CsA@F127DA/DA-PEG-GelMA multi-crosslinked, photocurable hydrogel for the treatment of DE. Through photocurable technology, this hydrogel can maintain the non-invasive and convenient nature of “eye-drop administration” while improving its adhesion to the cornea, achieving long-lasting adhesion to the ocular surface, significantly improving the retention time of the drug. This reduces the likelihood of drug flushing by tears and decreases the frequency of administration. The hydrogel is mainly composed of CsA@F127DA and DA-PEG-GelMA. F127 is prefunctionalized to have a double bond to form F127DA, allowing it to be photo-crosslinked in the presence of a photosensitizer (Fig. 1A). This hydrogel encapsulates the immunosuppressant CsA within F127DA and modifies GelMA using acryloyl PEG reactive ester via dopamine (DA), which possesses antioxidant properties (Fig. 1, Fig. 1C). Upon exposure to 405 nm light, the hydrogel catalyzes the chain addition reaction of double bonds on the surface of F127DA and GelMA, facilitating polymerization and self-assembly *in situ* to form a robust, multi-crosslinked network that offers benefits of anti-inflammatory and maintenance of redox homeostasis (Fig. 1D). Human corneal epithelial cells (HCECs) were used to establish a hyperosmotic stress model (500 mOsM) and a hydrogen peroxide oxidative stress model (200 μM) to evaluate the function of the hydrogels in scavenging ROS and maintaining mitochondrial redox homeostasis at cellular and subcellular level. The M1 polarization model of macrophages (RAW 264.7) was established and the M2 polarization level of cells was detected to evaluate the anti-inflammatory effect of the hydrogel at the cellular level. The efficacy and mechanism of the hydrogel in alleviating DE symptoms were further evaluated in a mouse DE model. CsA@F127DA/DA-PEG-GelMA can improve corneal epithelial glucose metabolism, maintain mitochondrial redox homeostasis and reduce oxidative stress injury by inhibiting the HIF-1α signaling pathway. In addition, it can inhibit the NF-κB signaling pathway and conjunctival inflammatory infiltration, finally restore tear secretion, relieve DE symptoms, and achieve efficient and safe treatment. The hydrogel has demonstrated remarkable biosafety and substantial therapeutic efficacy in treating DE, offering fresh perspectives on the management of other ocular diseases involving oxidative stress and inflammation.

**Fig. 1:**
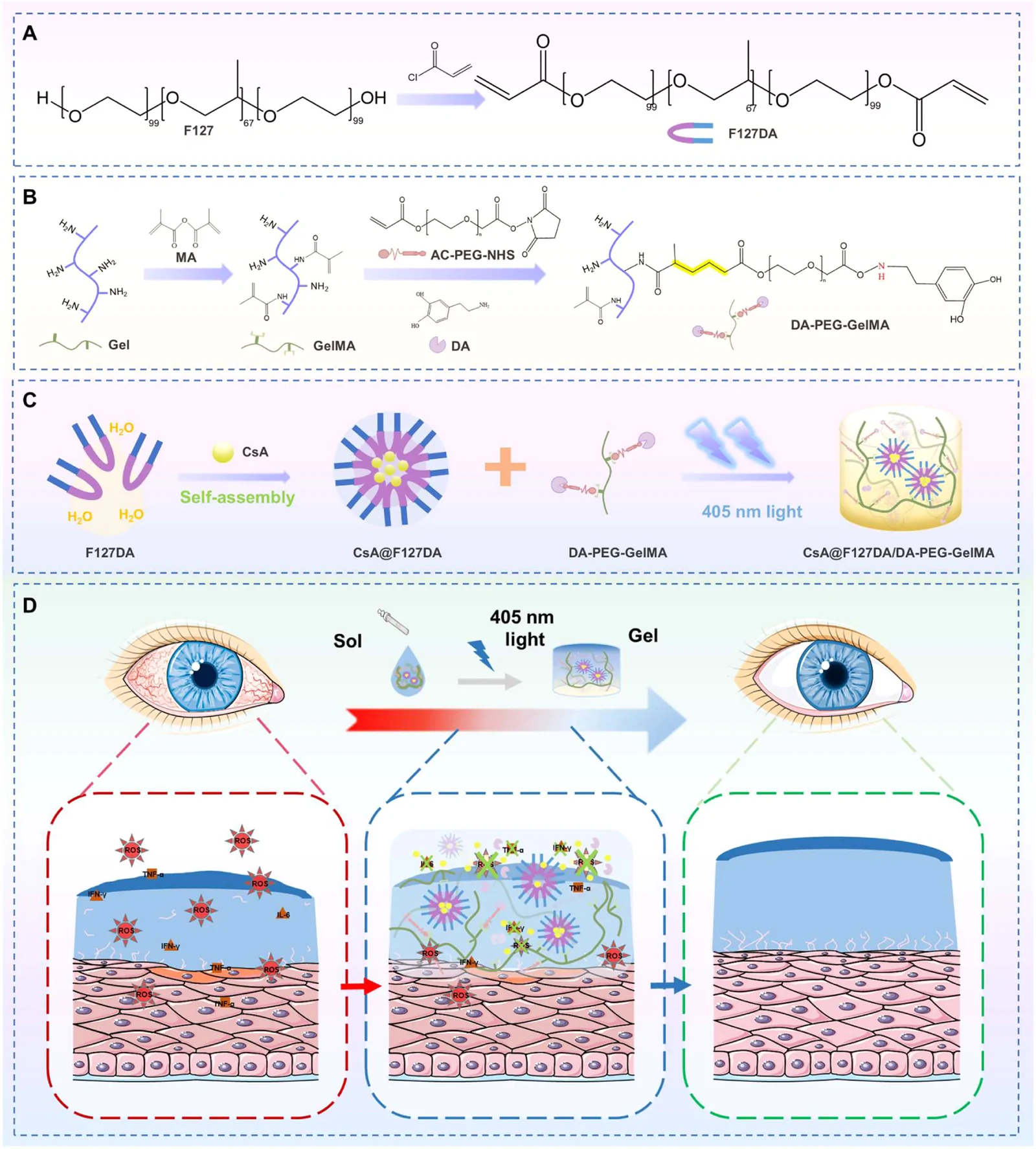
Illustrative scheme of the synthesized CsA@F127DA, DA-PEG-GelMA and CsA@F127DA/DA-PEG-GelMA for dry eye. (A) Synthesis of F127DA. (B) Synthesis of GelMA and DA-PEG-GelMA. (C) Synthesis of CsA@F127DA and CsA@F127DA/DA-PEG-GelMA. (D) Description of engineered ROS suppression and anti-inflammatory, breaking the vicious cycle of dry eye by loading CsA with multi-crosslinked photocurable hydrogels to improve ocular symptoms.

## 2 Materials and methods

### 2.1 Materials

Pluronic F127 (F127) and DA were obtained from Shanghai Macklin (shanghai, China). CsA was sourced from Boston TargetMol (MA, USA). Lithium phenyl-2,4,6-trimethylbenzoylphosphinate (LAP) and AC-PEG-NHS were sourced from Suzhou EFL (Suzhou, China). PBS, paraformaldehyde, periodic acid-Schiff (PAS) staining lit, oil red staining kit were from Solarbio (Beijing, China). Chloroform and isopropanol were from Sinopharm (Shanghai, China). Cell counting kit-8 (CCK8), crystal violet staining, Triton and 2′,7′-dichlorofluorescein diacetate (DCFH-DA) were from Beyotime (Shanghai, China). Live/dead cell assay was from Yeasen (Shanghai, China). Anti-SOD1 (1:200) and anti-SOD2 (1:200) were from Proteintech (Wuhan, China). RAW 264.7 cells were purchased from ATCC and RAW 264.7 specific medium was from Procell (Wuhan, China). Anti-CAT (1:200), anti-NOS2 (1:200), anti-CD86 (1:200), anti-TOM20 (1:200), Alexa Fluor 488-conjugated goat anti-rabbit IgG (1:300), anti-HO-1 (1:200), anti-CAT (1:200) and anti-IL-1β (1:200) were from Abclonal (Wuhan, China). Anti-Arg1 (1:200), anti-MMP3 (1:200) and anti-MUC5AC (1:200) were from Immunaway (Wuhan, China). Anti-CD206(1:200) was from Abcam (Cambridge, UK). Hematoxylin-eosin (H&E) staining kit was from Biosharp, TdT-mediated dUTP nick-end labeling (TUNEL) staining was from Servicebio (Wuhan, China) and LipidTOX neutral lipid stain was from Thermo Fisher Scientific Inc. Carbomer gel and tropicamide eye drops were obtained from Bausch & Lomb Corp. (Vaughan, Canada)

### 2.2 Synthesis and preparation

F127 and triethylamine were dissolved in dichloromethane, then acryloyl chloride was added, and the reaction was continued at 25 °C for 24 h after 1 h reaction in an ice water bath. At the end of the reaction, the triethylamine hydrochloride precipitate was removed by filtration. The filtrate was washed with saturated Na_2_CO_3_ aqueous solution and NaCl solution respectively, and then the layers were separated by standing, and the dichloromethane layer was taken. After drying the organic phase, it was concentrated using a rotary evaporator and dried under vacuum to obtain F127DA [19]. CsA (1 mg) was dissolved in anhydrous ethanol to ensure uniform dissolution, which effectively enhance the solubility CsA. Dissolved F127DA (100 mg) in anhydrous ethanol mixd with CsA solution and then ultrasonicate. Then 0.25% LAP (1 ml) was added slowly dropwise during ultrasonication for 30 min and stirred for 24 h away from light. Before photocrosslinking, the CsA@F127DA precursor solution was further diluted one-fold with 0.25% LAP solution to obtain the CsA@F127DA working solution.

Gelatin was dissolved in carbonate-bicarbonate buffer solution (CBS) and stirred at 50 °C until complete dissolution. The pH was adjusted to 9 using NaOH solution, and methacrylic anhydride (MA) was added dropwise, keeping the pH at 9. After 3 h of reaction, the pH was adjusted to 7.4 using HCl solution, and the impurities were removed by dialysis for 7 d, and lyophilized to obtain GelMA [17].

DA (4 mg) and AC-PEG-NHS (80 mg) were individually dissolved in 0.25% LAP (500 μl), then combine the solutions and allow them to mix thoroughly at 4 °C overnight away from light. GelMA (200 mg) was dissolved in 0.25% LAP (1 ml) and heated to 40 °C in a bath. DA-PEG-GelMA was obtained by mixing DA-PEG-NHS with GelMA in equal volumes. Before photocrosslinking, the DA-PEG-GelMA precursor solution was further diluted one-fold with 0.25% LAP solution to obtain the DA-PEG-GelMA working solution. CsA@F127DA precursor solution was mixed with equal volumes of DA-PEG-GelMA precursor solution to obtain CsA@F127DA/DA-PEG-GelMA. GelMA (200 mg) was dissolved in 0.25% LAP (2 ml) and heated to 40 °C in a bath, and F127DA (100 mg) was dissolved in LAP (1 ml). The GelMA and F127DA solutions were mixed in equal volumes to obtain F127DA/GelMA.

### 2.3 Transparency and transmittance analysis

CsA@F127DA, DA-PEG-GelMA and CsA@F127DA/DA-PEG-GelMA were individually introduced into vials, with their states documented through photography using a digital camera. Subsequent to photo-curing with 405 nm light, additional photographs were captured to facilitate a comparison of the hydrogel transparency across different vials. Furthermore, droplets of CsA@F127DA, DA-PEG-GelMA and CsA@F127DA/DA-PEG-GelMA were placed onto a letter card and recorded with a digital camera. Post-photocuring under 405 nm light, the transparency of these hydrogels was reassessed through photography.

In addition, separate cuvette containing CsA@F127DA, DA-PEG-GelMA and CsA@F127DA/DA-PEG-GelMA was subjected to photocuring under 405 nm light. This cuvette was then inserted into a UV–visible spectrophotometer (cary-60, Ajilent, Santa Clara, California, US) and scanned within the wavelength range of 200–800 nm to quantify its transmittance. Using Origin software, a transmittance curve was subsequently plotted.

### 2.4 Hydrogel formation performance testing

CsA@F127DA, DA-PEG-GelMA and CsA@F127DA/DA-PEG-GelMA were added into separate vial, photocured under 405 nm light, and subsequently placed at an inclined position. The photographic documentation was captured utilizing a digital camera for the purpose of observing the photocurable effect exhibited by the hydrogels.

### 2.5 In vitro degradation tests

CsA@F127DA, DA-PEG-GelMA and CsA@F127DA/DA-PEG-GelMA were poured into the molds and subjected to photocuring under 405 nm light. Then the hydrogels were carefully extracted and submerged in PBS to ensure complete absorption. After soaking for 48 h, any residual liquid on the hydrogel surface was removed with absorbent paper, and the initial mass of the hydrogel was recorded. Subsequently, the hydrogel was placed back into PBS, with daily weighing conducted at a consistent time, and the PBS solution was replaced with a fresh one each day. The hydrogels were immersed in a PBS solution containing collagenase, and after a specified interval, they were weighed again to estimate their degradation rate under physiological conditions. The calculation formula is as follows: Mass remained (%) = (W_i_−W_d_)/W_d_ × 100%, where W_d_ is the initial dry mass of the hydrogel, and W_i_ is the mass of the hydrogel at time *d*.

### 2.6 Swelling ratio and water content detection

CsA@F127DA, DA-PEG-GelMA and CsA@F127DA/DA-PEG-GelMA were taken and placed into a suitable mold and then photocured using 405 nm light. The hydrogels were removed and placed in PBS solution to fully swell. They were weighed and recorded at intervals until swelling equilibrium (i.e., the difference between two consecutive weight measurement was less than 0.01 g). After the excess liquid on the hydrogel surface was wiped off with absorbent paper, the mass was weighed with a precision balance as the initial mass Wp. The hydrogel was freeze-dried with a freeze-dryer and weighed again with a precision balance, and the mass was recorded as We. The swelling rate was calculated as follows:

Swelling rate (%) = (Wp-We)/Wd × 100%.

Water content of the hydrogel (%) = (Wp -We)/Wp × 100%.

### 2.7 Environmental SEM and TEM observation

The microscopic morphology and pore structure parameters of the hydrogels were analyzed using an environmental scanning electron microscope (SEM, Quanta 450). Cellular ultrastructure was observed by transmission electron microscopy (TEM). The hydrogel samples were snap-frozen (Quorum PP3000T Cryopreparation and Transport System), fractured, sublimated and gold-plated, and sent to the environmental SEM sample chamber for observation. For TEM analysis of cells, the cell samples were fixed, dehydrated, embedded, sectioned into ultrathin slices (70–90 nm), negatively stained, and then examined under TEM.

### 2.8 TGA and compression strength analysis

Thermogravimetric analysis (TGA) of hydrogel samples was performed in the TGA4000 (PE) analyzer. The samples were heated from room temperature to 500 °C at a rate of 10 °C/min with a nitrogen flow rate of 60 ml/min and the thermal stability was recorded. TGA curves were plotted using Origin software.

Cylindrical samples of CsA@F127DA, DA-PEG-GelMA, CsA@F127DA/DA-PEG-GelMA hydrogels were prepared with a diameter of 1 cm and a height of 1 cm. An external force or load was applied and the stress and strain relationship of the hydrogel was examined using an electronic universal material testing machine. Stress is the force per unit area, and strain is the degree of deformation of a material under the action of an external force. The stress-strain curves were plotted using Origin software.

### 2.9 FTIR spectroscopy, DLS and zeta potential detection

CsA@F127DA, DA-PEG-GelMA, CsA@F127DA/DA-PEG-GelMA and pure KBr were ground uniformly and pressed into transparent thin sheets. The spectra were collected by Fourier transform infrared (FTIR) spectrometer (FITR-650) and analyzed by OMNIC (Thermo Fisher Scientific Inc., Waltham, MA, USA) software.

The size of micelles of F127DA and CsA@F127DA in solution was detected using a dynamic light scattering (DLS) particle size analyzer (Zetasizer Nano ZS-90, Malvern Panalytical, Malvern, Worcestershire, Britain). The solvent LAP was used to dilute F127DA and CsA@F127DA in the ratio of 1:100 and 1 mL was taken for the detection. An electric field was applied to induce electrophoretic movement of the charged particles to further measure the Zeta potential of the sample particles.

### 2.10 Rheological behavior analysis

The flow and deformation properties of samples were tested via rotational rheometer (MCR302, Anton Paar, Graz, Austria). Rheological measurements were carried out using a PP-25 plate. The temperature was set from 4 °C to 60 °C and the heating rate was 5 °C/min to detect the temperature at which the hydrogel deformed. The rheological properties of the hydrogel were investigated by irradiating the hydrogel with 405 nm light at 75 s to 100 s.

### 2.11 Drug release tests

The release of CsA from CsA@F127DA/DA-PEG-GelMA hydrogel was evaluated by dialysis method. 20 μl CsA@F127DA and CsA@F127DA/DA-PEG-GelMA hydrogel were added to the bottom of a 15 ml centrifuge tube (Vazyme, Nanjing, China) and photocured with 405 nm light. After gel formation, 10 ml PBS containing 0.5% (w/v) Tween 80 was added as a release medium. The system was incubated in a thermostatic shaking incubator at 37 °C with a shaking speed of 200 rpm. At predetermined time intervals, 100 μl of release medium was withdrawn and replenished with a equal volume of fresh release medium. CsA in the release medium was determined by High Performance Liquid Chromatograph-Mass Spectrometer (HPLC-MC) (Waters Xevo TQ Absolute, Waters, Milford, MA, USA).

### 2.12 HPLC-MS examination

HPLC-MS analysis was performed on a C_18_ column (2.1 mm × 100 mm, 2.1 µm) at 60 °C. Mobile phase consisted of acetonitrile and 0.1% formic acid aqueous solution with gradient elution. Flow rate: 0.9 ml/min. Mass spectrometry was performed in electrospray positive ionization (ESI⁺) mode with multiple reaction monitoring (MRM). Quantification was carried out using extracted ion chromatograms, and the calibration curve was constructed with a series of standard solutions.

### 2.13 In vivo imaging examination

After anesthesia, the hydrogel eye drop was applied onto the ocular surface of the mice. The retention of the hydrogel on the ocular surface was recorded at different time points using an *in vivo* imaging system (IVIS Spectrum, PerkinElmer, Waltham, MA, USA).

### 2.14 Cell culture

HCECs were obtained from American Type culture Collection (ATCC). HCECs were cultured at 37 °C in an incubator containing 5% CO_2_ in a medium consisting of DMEM/F12, 6% fetal bovine serum, 7.5 µg/ml insulin, 10 ng/ml human epidermal growth factor and 1% penicillin/streptomycin. The hyperosmolar stress (HS) model was derived from hypertonic (500 mOsM) medium with DMEM/F12 and NaCl. The hydrogen peroxide (H_2_O_2_) oxidative stress model was developed by incubating cells with DMEM/F12 containing 200 µM H_2_O_2_ for 6 h. RAW 264.7 cells were cultured in an incubator with 5% CO_2_ using RAW 264.7 specific medium, consisting of DMEM, 10% fetal bovine serum and 1% penicillin/streptomycin.

After photocuring the hydrogel solution for 30 s at the bottom of the culture plate, a serum-free blank medium was added to guarantee thorough absorption of the hydrogel. Following this, the blank medium was discarded, and the cell suspension was introduced. Subsequently, the cells were periodically photographed for observation purposes.

### 2.15 In vitro biocompatibility tests

Cytotoxicity was evaluated by CCK8 and live/dead cell assay, and cell proliferation was evaluated by crystal violet staining . Leachate needs to be prepared before the experiment: culture medium was added to the hydrogels and immersed for 24 h to simulate the circulation of body fluids within the human body. When the cell growth density reached 70%–80%, the old medium was removed and the cells were cultured using the leachate from different hydrogels for 12 h or 24 h. Live/dead cell staining and crystal violet staining were performed after removing the medium. Alternatively, medium containing 10% CCK8 detection reagent was added, and the OD value was detected by microplate reader (Bio Tek Instruments, Winooski, VT) after incubated in the incubator for 2 h.

### 2.16 qRT-PCR

Prior to quantitative real-time polymerase chain reaction (qRT-PCR), cells or tissues were lysed by Trizol (TaKaRa, Shiga, Japan). Chloroform was added, and the mixture was centrifuged. The resulting supernatant was collected, mixed with isopropanol, and incubated at −80 °C for 30 min to precipitate total RNA. The RNA pellet was washed with ethanol. Reverse transcription was performed using the HiScript III All‑in‑one RT SuperMix Perfect for qPCR (Vazyme) to synthesize cDNA. Quantitative PCR was carried out on a LightCycler system (Roche, Basel, Switzerland). The expression level of each gene was calculated using β-actin as an internal reference. The primer information is shown in Table 1, Table 2.

**Table 1:** Primers for qRT-PCR (Mouse).

Gene	Forward primer sequence (5'-3')	Reverse primer sequence (5'-3')
β-actin TNF-α MMP3 NOS2 CD206 IL-10 IL-13 SOD1 SOD2 CAT HO-1 IL-17 IL-6	CCTAAGGCCAACCGTGAAAAG TGTAGCCCACGTCGTAGCAA CCTTTTGATGGGCCTGGAAC GCCCAGCCAGCCCAAC GTCAGAACAGACTGCGTGGA GCTCCAAGACCAAGGTGTCT GCAGCATGGTATGGAGTGT GTCGGCTTCTCGTCTTGCTC AGGAGAGTTGCTGGAGGCTA AGGCTCAGCTGACACAGTTC CCTCACAGATGGCGTCACTT CGCAATGAAGACCCTGATAGAT CCCCAATTTCCAATGCTCTCC	AGGCATACAGGGACAGCACAG AGGTACAACCCATCGGCTGG GAGTGGCCAAGTTCATGAGC GCAGCTTGTCCAGGGATTCT AGGGATCGCCTGTTTTCCAG AGGACACCATAGCAAAGGGC TATCCTCTGGGTCCTGTAGATG GCTTTCATCGCCATGCTTCC TAGTAAGCGTGCTCCCACAC ATGGAGAGACTCGGGACGAA TGGGGGCCAGTATTGCATTT CTCTTGCTGGATGAGAACAGAA CGCACTAGGTTTGCCGAGTA
IL-1β	GGGCCTCAAAGGAAAGAATC	TACCAGTTGGGGAACTCTGC
IFN-γ	AAATCCTGCAGAGCCAGATTAT	GCTGTTGCTGAAGAAGGTAGTA
MUC5AC	ATGGGCTGTGTTCCTGTGTC	CAGAACATGTGTTGGTGCAGTC

**Table 2:** Primers for qRT-PCR (Human).

Gene	Forward primer sequence (5'-3')	Reverse primer sequence (5'-3')
β-actin	ACAGAGCCTCGCCTTTGC	GCGGCGATATCATCATCC
SOD1	GGTGGGCCAAAGGATGAAGAG	CCACAAGCCAAACGACTTCC
SOD2	GCTCCGGTTTTGGGGTATCTG	GCGTTGATGTGAGGTTCCAG
CAT	TGGAGCTGGTAACCCAGTAGG	CCTTTGCCTTGGAGTATTTGGTACC
NRF2	TCAGCGACGGAAAGAGTATGA	ACTGGTTTCTGACTGGATGT

### 2.17 Immunofluorescence staining

Cells were fixed using 4% paraformaldehyde after treated with different formulations, then washed with PBS, blocked with BSA solution, and permeabilized using Triton. Anti-SOD1 (1:200), anti-SOD2 (1:200), anti-CAT (1:200), anti-NOS2 (1:200), anti-CD86 (1:200), anti-Arg1 (1:200), anti-CD206(1:200) and anti-TOM20 (1:200) were incubated overnight then incubated with Alexa Fluor 488-conjugated goat anti-rabbit IgG (1:300) for 1 h away from light. The slices were securely sealed with a DAPI-infused sealer (Vector, Burlingame, CA, USA), subsequently photographed, and meticulously documented through fluorescence microscopy (Leica DM2500, Leica, Wetzlar, Germany).

After mice were euthanatized, the whole eyeballs of mice were removed. Paraffin sections were obtained after dehydration, transparency, embedding and sectioning. Before staining, paraffin sections were dewaxed, rehydrated [20]. After incubated with Triton buffer and blocked with BSA solution, anti-SOD1 (1:200, Proteintech), they were incubated overnight with anti-HO-1 (1:200), anti-CAT (1:200), anti-MMP3 (1:200), anti-MUC5AC (1:200), anti-IL-1β (1:200), anti-CD86 (1:200) and anti-CD206 (1:200), then incubated with Alexa Fluor 488-conjugated goat anti-rabbit IgG (1:300) for 1 h away from light. Slices were covered using DAPI-containing blocking agent (Vector), finally, photographed and documented by fluorescence microscopy (Leica DM2500).

### 2.18 Flow cytometry tests

Cells were co-incubated with hydrogels of various formulations in either H_2_O_2_ or HS solution. Following this, the cells were thoroughly washed multiple times and subsequently incubated in medium containing DCFH-DA. After incubation, the cells were washed and resuspended in PBS, filtered through a flow-through tube strainer (Biosharp, Beijing, China), and gently flicked to ensure thorough mixing. Intracellular ROS levels of the collected cells were then measured using flow cytometry. The resulting data were exported and analyzed utilizing FlowJo software. Separately, RAW 264.7 cells were seeded into 12-well plates at a density of 2 × 10^5^ cells per well. These cells were pretreated with LPS (100 ng/ml) and IFN-γ (20 ng/ml). And the cells were incubated for 24 h in a blank medium containing hydrogels of different formulations. Afterward, the cells were collected, and the level of transformation of RAW 264.7 cells ware assessed by flow cytometry, by detecting the expression levels of CD86. The obtained data were exported and analyzed by FlowJo software.

### 2.19 DE murine model establishment

Female C57BL/6 mice (6–8 w, body weight 20 ± 5 g) were purchased from Shanghai SLAC Co. All animal experiments have complied with the Association for Research in Vision and Ophthalmology Statement for the Use of Animals in Ophthalmic and Vision Research and the guidelines for Animal Care and Use Committee of the university of South China. All animal experiments were approved by the Animal Ethics Committee of the University of South China (Approval No. 2024-241). The mice were housed in a standard environment at constant temperature (22 ± 1 °C) with regular light/dark cycles (12 h/12 h), free access to food and water, and standard dietary feeding. BAC-dependent experimental DE mice model was established. Briefly, 2 µl of 0.075% BAC (Sigma-Aldrich, Saint Louis, MO, USA) eye drops (w/v) were spotted in both eyes of each mouse twice a day for 7 d. Then the mice were randomly divided into 5 groups and were given 2 µl one of the following formulations: CsA@F127DA, DA-PEG-GelMA, CsA@F127DA/DA-PEG-GelMA, F127DA/GelMA or Restasis®, respectively once a day. CsA@F127DA, DA-PEG-GelMA and CsA@F127DA/DA-PEG-GelMA were cured using 405 nm light, applied for 5 s at a distance of 30 cm lasting 5 s after eye drops administration.

### 2.20 Tear secretion tests

The tear secretion of mice was detected using a phenol red cotton thread (Tianjin Jingming New Technology Development Co., Ltd., Tianjin, China). Grasp the phenol red cotton thread and carefully position it onto the conjunctival vault of the mice. The length of the phenol red cotton thread, which had been wetted and reddened by the tear, was measured in millimeters, and the eyes were manually transiently eyed after the completion of the test to avoid overexposure.

### 2.21 Corneal fluorescein staining

Corneal epithelial damage in mice was detected by slit-lamp fluorescence staining. After successfully administering anesthesia to the mice, their eyeballs were fully exposed. The cornea was then thoroughly cleaned using saline solution. With the cornea positioned perpendicular to the light source, an image was captured under the bright white light of the slit lamp. Subsequently, 2 µl fluorescein sodium (1%) was instilled, followed by manual rinsing of the eyes several times with PBS. To eliminate any excess fluid and fluorescein staining from the eye surface and eyelid periphery, filter paper was utilized, ensuring complete exposure of the eye. Finally, under 405 nm light conditions with the slit lamp, a detailed image of the fluorescein staining of the corneal epithelium was captured. The cornea was scored for fluorescein staining according to a standard 12-point scale: the cornea was divided into 4 quadrants, with each quadrant scored 0–3 points; no staining was scored as 0 point; 1–30 punctate stains was scored as 1 point; greater than 30 punctate stains with no fusion of stains was scored as 2 points; and the presence of punctate stains in the cornea with fusion, filaments, or ulcers was scored as 3 points.

### 2.22 In vivo confocal microscope examination

Corneal epithelial damage was assessed through *in vivo* confocal microscope. After anesthetizing the mice, their corneas were meticulously cleaned with saline. With the eyes fully exposed, a layer of carbomer gel was applied to the cornea, ensuring that the cornea remained perpendicular to the light source. The *in vivo* confocal microscope was then adjusted to various focal lengths to capture images that detailed the tissue structure of the cornea at multiple depths.

### 2.23 Optical coherence tomography examination

After anesthetizing the mice and the pupils were dilated with tropicamide eye drops, the eyes were fully exposed, and the position and angle of the mice were adjusted to keep the cornea perpendicular to the light source and the pupil facing the light source. Optical coherence tomography (OCT) images of the cornea and retina were captured by an OCT scanner (HealthOlight Technology Co., Ltd. Beijing, China), and the central thickness of the cornea and retina was measured and counted.

For quantitative analysis of corneal thickness, OCT cross-sectional images were analyzed using ImageJ software. Corneal thickness was measured at three predefined regions, including the central corneal region and two peripheral corneal regions. Three eyes from each experimental group were randomly selected for analysis, and three independent measurements were obtained from each eye. The mean value of these measurements was calculated and used for statistical analysis.

### 2.24 Fundus photography and fundus fluorescein angiography examination

Fundus photography was utilized to conduct comprehensive imaging examinations of the mouse retina. For 7 d consecutivly, various aqueous gel eye drop formulations were administered once daily. Then the mice were fully anesthetized, and their pupils were dilated using tropicamide drops. Using the OPTOPROBE system (HealthOlight Technology Co., Ltd.) for fundus photography, detailed images of each eye’s fundus were captured. With the mice still anesthetized and their pupils dilated, the optic nerve was centered, and the retinal vessels were meticulously photographed and recorded. Subsequently, 0.01 ml of 10% sodium fluorescein, a contrast agent, was injected intraperitoneally. Once the contrast agent had circulated to the fundus blood vessels, the retinal blood vessels were photographed and recorded using blue wavelength light excitation.

### 2.25 H&E, PAS and TUNEL staining

After mice were euthanized, whole eyeballs of mice were removed, excess tissues were removed and the eyeballs were fixed using 4% paraformaldehyde. The paraffin sections were dewaxed and rehydrated before staining. Staining was performed using a H&E staining kit according to the instructions, and imaging was performed by light microscopy (Nikon, Melville, NY, USA). The PAS Staining Kit was performed following the manufacturer’s instructions. Images were captured by light microscope (Nikon). The number of PAS-stained conjunctival goblet cells (GCs) was counted and statistically analyzed.

TUNEL staining was performed according to the manufacturer’s instructions. After slides were covered with DAPI, a fluorescence microscopy system (Leica DM2500, Leica) was used to capture images. The TUNEL positive cells were counted by ImageJ software.

### 2.26 Oil red staining and LipidTOX staining

Oil red staining kit and LipidTOX neutral lipid stain were involved according to the manufacturer’s instructions. After staining, images were captured by light microscope (Nikon), and lipids of lacrimal gland and meibomian gland were quantified by ImageJ software.

### 2.27 Statistical analysis

For statistical analysis using GraphPad Prism software (GraphPad software, San Diego, CA, USA), *P* < 0.05 was set as the threshold of significance level for comparison between two groups. For parameter tests among multiple sets of data, one-way analysis of variance (ANOVA) or Welch’s analysis of variance combined with Dunnett’s test was used, with results presented as mean ± standard error of the mean (S.E.M). For nonparametric tests, the Kruskal-Wallis test was used, with results presented as median ± interquartile range (IQR).

## 3 Results and Discussion

### 3.1 Preparation and characterization of CsA@F127DA, DA-PEG-GelMA and CsA@F127DA/DA-PEG-GelMA

FTIR spectroscopy was employed to characterize Gel and GelMA. GelMA exhibits noticeable changes in the absorption bands around 1445 cm⁻¹ and 1236 cm⁻¹ compared with Gel, which can be attributed to variations in alkyl and C–O related vibrations induced by MA. Meanwhile, the characteristic amide I (∼1624 cm⁻¹) and amide II (∼1534 cm⁻¹) bands remain present, indicating that the gelatin backbone is preserved after modification. These spectral features are consistent with previously reported FTIR characteristics of GelMA, suggesting the successful introduction of MA functionalities (Fig. S1). In addition to FTIR characterization, LC–MS analysis was further performed to evaluate the possible presence of residual small-molecule reagents or impurities in the purified GelMA and F127DA samples. The LC–MS results showed no detectable signals corresponding to the relevant residual small-molecule reagents. The synthesis of CsA@F127DA, DA-PEG-GelMA and CsA@F127DA/DA-PEG-GelMA was performed. Additionally, the chemical structures of the products were characterized by ¹H nuclear magnetic resonance (¹H NMR) spectroscopy. In the ¹H NMR spectrum of DA-PEG-NHS, characteristic proton signals originating from the aromatic ring of DA were clearly observed. Meanwhile, in the spectrum of AC-PEG-NHS, the characteristic signals corresponding to the vinyl protons of the acryloyl group were detected. The presence of these characteristic signals indicates the successful introduction of the corresponding functional groups, further confirming that DA was successfully grafted onto the PEG backbone (Fig. S2). The formation of special peaks in FTIR spectra proved the successful synthesis of DA-PEG-GelMA and CsA@F127DA/DA-PEG-GelMA. Benzene ring C-H bond stretching vibrations in DA, DA-PEG-GelMA and CsA@F127DA/DA-PEG-GelMA were observed with overlapping absorption bands near 3033 and 3061 cm^−1^. N-H and O-H in GelMA, DA-PEG-GelMA and CsA@F127DA/DA-PEG-GelMA produced a moderate absorption band near 3282 cm^−1^. The absorption peak of C = O often appears in 1670–1755 cm^−1^, but its position can be shifted by the influence of the environment. The absorption peaks of C = O for GelMA and DA-PEG-GelMA appear around 1633 cm^−1^. The absorption peaks of C = O for F127DA and CsA@F127DA/DA-PEG-GelMA appear around 1750 cm^−1^ (Fig. 2A).

**Fig. 2:**
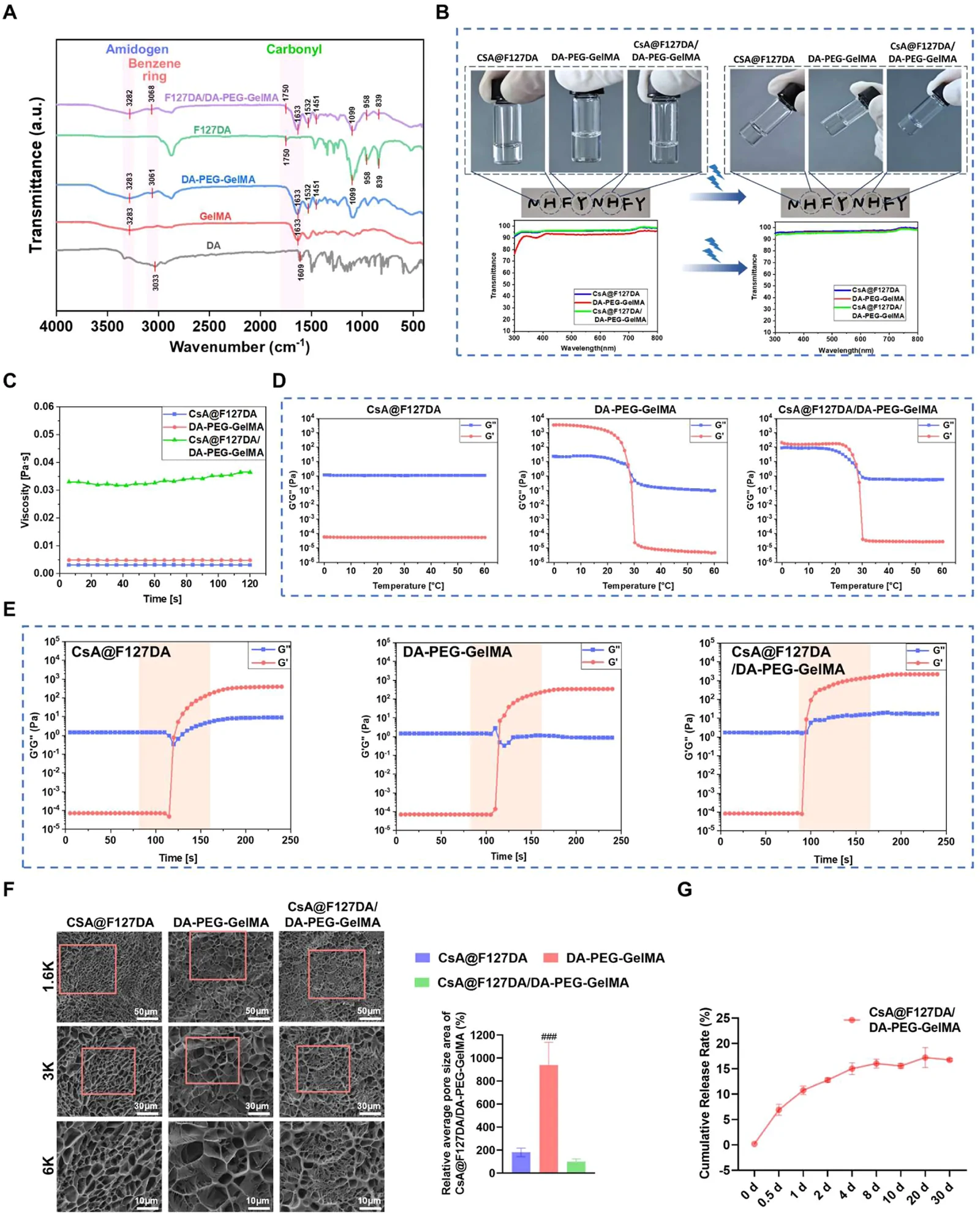
Characterization of CsA@F127DA, DA-PEG-GelMA and CsA@F127DA/DA-PEG-GelMA. (A) FTIR spectra of DA, GelMA, DA-PEG-GelMA, F127DA and CsA@F127DA/DA-PEG-GelMA; (B) Transparency and transmittance between 300 nm to 800 nm of CsA@F127DA, DA-PEG-GelMA and CsA@F127DA/DA-PEG-GelMA; (C) Viscosity of CsA@F127DA, DA-PEG-GelMA and CsA@F127DA/DA-PEG-GelMA; (D) Rheological properties of CsA@F127DA, DA-PEG-GelMA and CsA@F127DA/DA-PEG-GelMA at 0–60 °C and (E) under 405 nm light irradiation; (F) Microstructure and pore size statistics of CsA@F127DA, DA-PEG-GelMA and CsA@F127DA/DA-PEG-GelMA under Cryo-SEM (*n* = 3 per group). (G) CsA release curve of CsA@F127DA/DA-PEG-GelMA in 0.5% PBST. Data are expressed as mean ± S.E.M., ^###^*P* < 0.001. ^#^Points to a statistical comparison between this group and CsA@F127DA/DA-PEG-GelMA group.

Transparency serves as a crucial physical metric for assessing the suitability of hydrogels for ocular surface applications. Achieving optimal transparency is essential for maintaining clear vision following the administration of eye drops. To compare the light transmission capabilities of various hydrogels, their respective light transmission properties were measured. CsA@F127DA, DA-PEG-GelMA and CsA@F127DA/DA-PEG-GelMA were placed on a letter card. The word was clearly discernible on the bottom card for all three hydrogel groups, and the transparency of the hydrogels remained largely unchanged following photocrosslinking. The light transmission properties of different hydrogels were further quantified by UV-visible spectrophotometer. The results showed that in the visible light (390 nm–780 nm) wavelength range, the light transmittance of CsA@F127DA and CsA@F127DA/DA-PEG-GelMAwas greater than 95%, that of DA-PEG-GelMA was greater than 90%. After photocrosslinking, the transparency of CsA@F127DA, DA-PEG-GelMA and CsA@F127DA/DA-PEG-GelMA was greater than 95% (Fig. 2B).

The rheological test can provide valuable insights into the viscosity, modulus, and other key properties of hydrogels. The viscosities of CsA@F127DA, DA-PEG-GelMA and CsA@F127DA/DA-PEG-GelMA were examined by rheometer, and it was found that CsA@F127DA had the lowest viscosity and CsA@F127DA/DA-PEG-GelMA had the highest viscosity (Fig. 2C). Modulus changes of CsA@F127DA, DA-PEG-GelMA and CsA@F127DA/DA-PEG-GelMA at different temperatures and with 405 nm light irradiation were also detected. Temperature scan analysis in the temperature range of 4–60 °C reveals that CsA@F127DA exhibited a parallel trend between the loss modulus and the storage modulus in this temperature range, which implied that CsA@F127DA maintains liquid properties throughout the temperature range under investigation. This was related to the relatively low concentration of F127DA used in this test. At this relatively low polymer concentration, the micellar packing and entanglement density were insufficient to trigger a gelation process. It was particularly noteworthy that when the temperature was close to 30 °C, there was a significant intersection between the loss modulus and the storage modulus of DA-PEG-GelMA and CsA@F127DA/DA-PEG-GelMA, which is usually regarded as a key indicator of solid-liquid transition, indicating that material DA-PEG-GelMA and CsA@F127DA/DA-PEG-GelMA were undergoing a transformation process from liquid to solid or *vice versa* under these conditions (Fig. 2D). In addition, rheological analysis was used to systematically characterize the photo-response of material CsA@F127DA, DA-PEG-GelMA and CsA@F127DA/DA-PEG-GelMA. During the initial 50-s phase of 405 nm light-induced crosslinking, it was observed that the three hydrogels underwent a rapid gelation process. With the continuous extension of 405 nm light irradiation time, the storage modulus (G’) of these three hydrogels showed a significant increase trend. This observation may be due to the fact that the accumulation of light time and the enhancement of light intensity had a direct effect on the crosslinking degree of hydrogels, further leading to a significant evolution in the physical properties of the hydrogel. Taken together, these rheological characteristics revealed the complex mechanism of dynamic changes of structure and properties of photo-responsive hydrogels under 405 nm light irradiation. In addition, it is worth noting that CsA@F127DA/DA-PEG-GelMA exhibits a more rapid photocrosslinking property (Fig. 2E). To better characterize the CsA@F127DA/DA-PEG-GelMA’s resistance to deformation during the blinking process, we performed time scans at specific frequencies and strains. The results showed that CsA@F127DA/DA-PEG-GelMA exhibited good stability under high-frequency dynamic loading (Fig. S3).

The internal microstructure of photocrosslinked CsA@F127DA, DA-PEG-GelMA and CsA@F127DA/DA-PEG-GelMA was observed using Cryogenic SEM (Cryo-SEM) in detail. Cryo-SEM images clearly verify that photocrosslinked CsA@F127DA/DA-PEG-GelMA had a porous and reticular structure. Through comparative analysis, we found that compared with the other two hydrogels groups, the porosity of CsA@F127DA/DA-PEG-GelMA presented the minimum value (Fig. 2F). This phenomenon strongly suggested the existence of complex and multiple cross-linked structures within CsA@F127DA/DA-PEG-GelMA. In the field of materials science, the crosslinking density of hydrogels has a significant effect on their properties. In general, the more tightly the hydrogel is crosslinked, the slower the efficiency of drug release. In view of this, we further carried out the detection of the drug release curve for CsA@F127DA/DA-PEG-GelMA. The results showed that CsA@F127DA/DA-PEG-GelMA exhibited slower drug release properties (Fig. 2G). Furthermore, the insoluble CsA that was not encapsulated was removed by. The supernatant was then obtained and subjected to ultrasonic disruption. The results showed the encapsulation rate of CsA approximately 72.94% in CsA@F127DA through LC-MS. Further calculation reveals that the CsA loading capacity of CsA@F127DA was 0.72%. (Fig. S4). Besides, the released DA concentration in the extraction solution was quantitatively determined using HPLC. The results showed that the DA concentration in the 24 h extract was approximately 447.89 µg/mL in DA-PEG-GelMA and 294.79 µg/mL in CsA@F127DA/DA-PEG-GelMA (Fig. S5).

### 3.2 Properties of CsA@F127DA, DA-PEG-GelMA and CsA@F127DA/DA-PEG-GELMA

TGA was employed to assess the stability of hydrogels, mirror the thermal dynamic behavior of these hydrogels, and characterize and contrast the thermal properties among various hydrogels. The TGA curves showed that the thermal decomposition of CsA@F127DA, DA-PEG-GelMA and CsA@F127DA/DA-PEG-GelMA was divided into two stages. In the first stage, the weight of the hydrogel decreased rapidly, which corresponded to the loss of water in the hydrogel. The second stage was a slow decrease in the weight of the hydrogel, which corresponded to the decomposition of the compound (Fig. 3A). The compression properties of hydrogels were tested by a universal testing machine, and stress-strain curves were drawn to characterize and compare the compression modulus of hydrogels. It was observed that the sample CsA@F127DA/DA-PEG-GelMA showed the highest compressive modulus, which was preliminarily attributed to the highly dense crosslinked network structure in its interior (Fig. 3B). In order to evaluate the *in vitro* degradation properties of hydrogels and their stability in different environments, we focused on their performance in PBS and collagenase solutions. Through continuous and rigorous monitoring process, we found that in PBS, the quality of CsA@F127DA and CsA@F127DA/DA-PEG-GelMA did not change significantly, showing good stability. In contrast, the quality of the sample DA-PEG-GelMA showed a slight downward trend, although the change was not large (Fig. 3C). The degradation rate of DA-PEG-GelMA in collagenase was very fast, and the mass decreased by 63% after 1 h. CsA@F127DA was the most stable, with a mass reduction of only 26% after 24 h (Fig. S6). In addition, the swelling properties and water content of CsA@F127DA, DA-PEG-GelMA, CsA@F127DA/DA-PEG-GelMA were also tested. The results showed that CsA@F127DA/DA-PEG-GelMA presented the smallest swelling (Fig. S7). This phenomenon can be attributed to differences in the internal network structure. Similar phenomena have been widely reported, where hydrogel swelling behavior is governed not only by porosity but also by pore connectivity and internal network interactions. The pores in DA-PEG-GelMA are partially closed, which limits the diffusion of water molecules into the matrix [21]. The pores in DA-PEG-GelMA are mainly small or partially closed, which limits the diffusion of water molecules into the matrix. Moreover, the presence of dopamine and PEG segments introduces additional intermolecular interactions (*e.g.*, hydrogen bonding and π–π stacking), which restrict network expansion during swelling. Therefore, despite its higher porosity, the effective water absorption capacity of DA-PEG-GelMA is reduced, resulting in a lower swelling ratio. CsA@F127DA, DA-PEG-GelMA, CsA@F127DA/DA-PEG-GelMA all had a water content of more than 90% (Fig. S8).

**Fig. 3:**
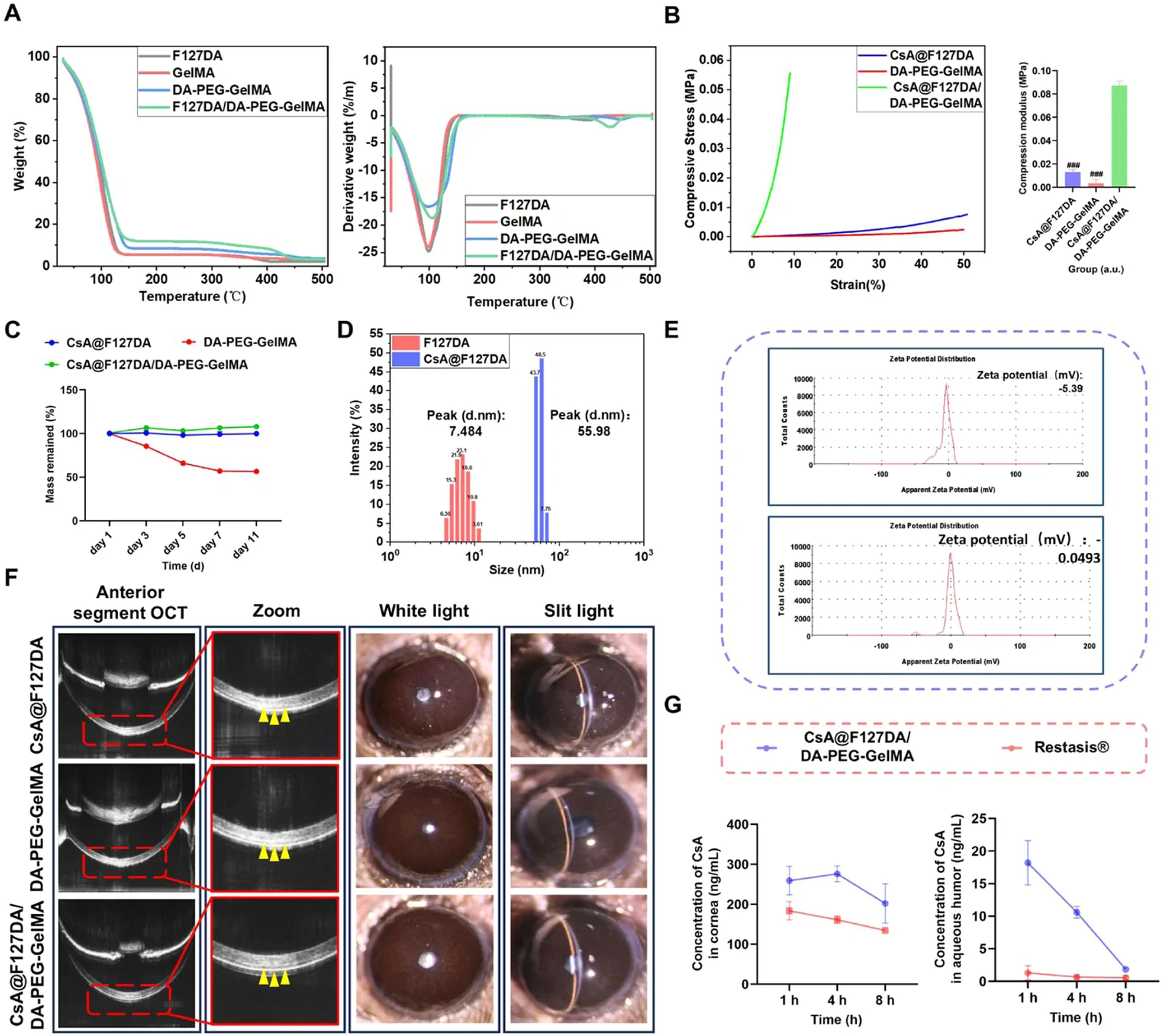
Properties of CsA@F127DA, DA-PEG-GelMA and CsA@F127DA/DA-PEG-GelMA. (A) TGA curves of DA, GelMA, DA-PEG-GelMA, F127DA and CsA@F127DA/DA-PEG-GelMA; (B) Stress-stain curves and compressive modulus of hydrogels during compression testes. (*n* = 3 per group); (C) Mass remained of hydrogels in PBS; (D) Particle size distribution of F127DA and CsA@F127DA micelles; (E) Zeta potential of F127DA and CsA@F127DA micelles measured by DLS; (F) OCT images and slit lamp of hydrogels on the ocular surface. (the yellow arrow indicates the hydrogel position on the cornea); (G) CsA concentrations in the cornea and aqueous humor 1 h, 4 h and 8 h after the use of CsA@F127DA/DA-PEG-GELMA and Restasis®. Data are expressed as mean ± S.E.M., ^###^*P* < 0.001. ^#^ Points to a statistical comparison between this group and CsA@F127DA/DA-PEG-GelMA group.

The self-assembly behavior of CsA@F127DA was evaluated by DLS. The results of DLS analysis clearly revealed that before the loading of CsA, the particle size distribution of F127DA was mainly concentrated at 7.484 nm. However, after CsA was successfully loaded, the particle size of CsA@F127DA complex formed increased significantly to 55.98 nm (Fig. 3D). In addition, the zeta potential value of CsA@F127DA was about −0.0493 mV. It is worth noting that compared with F127DA before loading, the potential value of CsA@F127DA increases and tends to the neutral charge state (Fig. 3E). This change not only reflected a possible interaction between CsA and F127DA but, more importantly, suggested that the risk of potential damage to corneal epithelial tissue from CsA@F127DA was extremely low. Upon application to the ocular surface, the gel state of the hydrogel on the cornea holds significant importance in determining its subsequent adhesion efficacy. A smooth and uniform gel morphology contributes to minimizing ocular discomfort experienced. Hence, we investigated the state of the hydrogel on the cornea by applying the liquid-state hydrogel, followed by manual blinking and photocrosslinking with 405 nm light. OCT was used to observe the distribution of hydrogels on the ocular surface of mice. Yellow arrows indicated the hydrogel adherence to the surface of the cornea. The results revealed that CsA@F127DA/DA-PEG-GelMA exhibited a superior gel-forming state upon being dropped onto the cornea, presenting a smoother and more uniform arcuate shape. This finding indicated that CsA@F127DA/DA-PEG-GelMA could provide enhanced comfort (Fig. 3F). To conduct a more comprehensive assessment of the *in vivo* pharmacokinetics of CsA@F127DA/DA-PEG-GelMA, HPLC-MS was employed to quantify the CsA concentrations in the rabbits’ cornea and aqueous humor of rabbits at 1 h, 4 h and 8 h post-administration of the eye drops. CsA content in cornea and aqueous humor of rabbits treated with CsA@F127DA/DA-PEG-GelMA was higher than that of commercially available CsA (Restasis®). Interestingly, after 4 h of treatment with CsA@F127DA/DA-PEG-GelMA, there was a small spike in CsA content in the cornea, which might be related to the slow-release effect of CsA@F127DA/DA-PEG-GelMA (Fig. 3G). The sustained retention of CsA@F127DA/DA-PEG-GelMA on ocular surface may be attributed to its DA modification technology. Given that DA is derived from bionic mussels, its excellent adhesion properties have been widely verified. Based on the molecular mechanism of mussel adhesion protein, DA can enhance the affinity of corneal mucin through multiple interactions such as catechol group-mediated π-π stacking, hydrogen bonding and metal chelation [22,23]. In addition to its excellent adhesive properties, DA also possesses good antioxidant capabilities. The results from the ABTS assays kit indicate that DA at concentrations of 1, 2 and 4 mg/ml all demonstrate strong antioxidant activity (Fig. S9).

### 3.3 Biocompatibility and safety of CsA@F127DA/DA-PEG-GelMA in vitro

To investigate the safety of hydrogels on HCECs, we extracted the leachate of hydrogels. The leachate arises from the ongoing exchange of substances between the material and the soaking solution, thus simulating the interaction that takes place between the material and biological tissues or bodily fluids within a living organism. In this study, the leachate of CsA@F127DA, DA-PEG-GelMA and CsA@F127DA/DA-PEG-GelMA in culture medium was extracted (Fig. S10A). HCECs were then cultured with this leachate for CCK8, crystal violet staining and live-dead cell staining. Cell viability after the use of the leachate was evaluated by CCK8 (Fig. S10B), the effect of the leachate on the live-dead of the cells was evaluated by live-dead cell staining (Fig. S11), and the effect of the leachate on the proliferative status of the cells was evaluated by crystal violet staining (Fig. S12). Cell viability was not significantly affected by incubation of HCECs with the leachates of CsA@F127DA, DA-PEG-GelMA and CsA@F127DA/DA-PEG-GelMA for 24 h and 48 h, respectively, and the crystalline violet and live-dead cell staining further proved the safety of the hydrogels. In order to further verify the non-invasive nature of the photopolymerization conditions on HCECs, we exposed the cells to irradiation for 10 s, 30 s, 60 s and 180 s, and quantitatively evaluated their viability by CCK-8 assay. The results indicated that, within the optimized exposure window (10 s–3 min), there is no significant reduction in cell viability (Fig. S13).

### 3.4 CsA@F127DA/DA-PEG-GelMA exerts antioxidant properties by protecting mitochondria under H_2_O_2_ oxidative stress

An imbalance in redox homeostasis plays a pivotal role in the progression of DE [24]. Intracellular ROS levels are used as important biomarkers to assess the level of oxidative stress. Upon exposure to unfavorable environments, ROS levels increase in HCECs. Excessive ROS can enhance ocular hypertonic stress and ocular surface inflammation, creating a vicious cycle. Therefore, removing excessive ROS from HCECs and maintaining the balance of cellular redox homeostasis are essential for the treatment of DE.

Consequently, to better validate the efficiency of hydrogels in reducing intracellular oxidative stress, we developed an oxidative stress model by exposing HCECs to H_2_O_2_ to mimic DE [25] (Fig. 4A). Transwell chambers were used to simulate the treatment of cells in the stress state by each group of hydrogels. The protection of HCECs viability by each group of hydrogels in the stress state was detected using the CCK8 method. The results showed that all three groups of hydrogels were able to rescue cell viability to a certain extent compared with the H_2_O_2_ group (Fig. 4B).

**Fig. 4:**
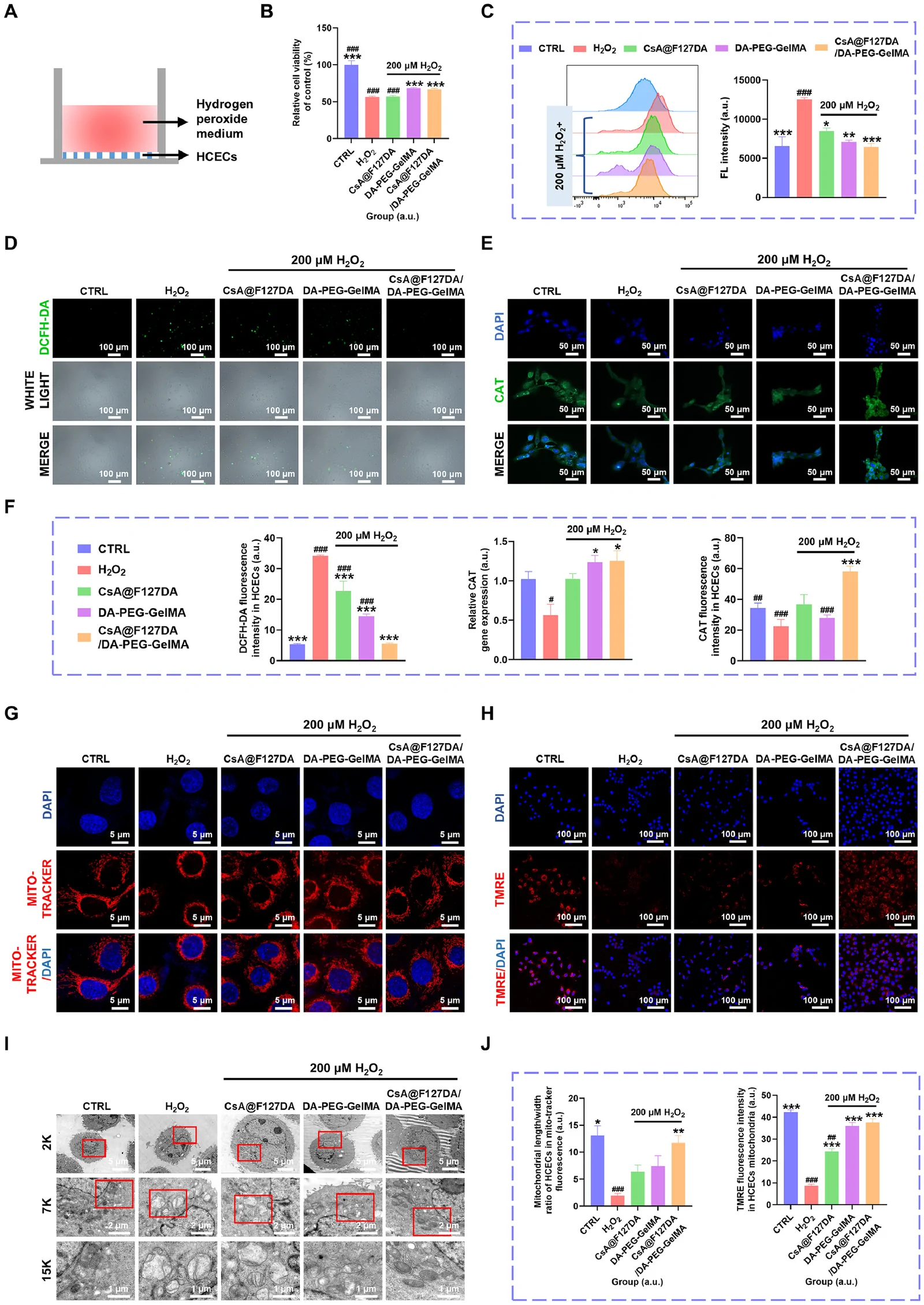
Antioxidant properties of CsA@F127DA/DA-PEG-GelMA under H_2_O_2_ oxidative stress. (A) Schematic representation of hydrogel action on HCECs model of H_2_O_2_ oxidative stress; (B) Relative cell viability of HCECs after H_2_O_2_ oxidative stress (*n* = 6 per group); (C) Quantification of ROS production levels in HCECs under H_2_O_2_ oxidative stress by flow cytometry (*n* = 3 to 4 per group); (D) Representative images of ROS live cells staining and (E) CAT fluorescence staining under H_2_O_2_ oxidative stress; (F) Fluorescence intensity quantification of ROS live cells staining and expression and fluorescence intensity quantification of CAT under H_2_O_2_ oxidative stress (*n* = 3 to 6 per group); (G) Representative images of Mito-Tracker staining, (H) TMRE staining and (I) mitochondrial TEM with different hydrogel treatments under H_2_O_2_ oxidative stress; (J) Fluorescence intensity quantification of Mito-Tracker and TMRE staining with different hydrogel treatments under H_2_O_2_ oxidative stress (*n* = 3 per group). Data are expressed as mean ± S.E.M., *P < 0.05, **P < 0.01, ***P < 0.001. ^#^*P* < 0.05, ^##^*P* < 0.01, ^###^*P* < 0.001. *Points to a statistical comparison between this group and H_2_O_2_ group. ^#^Points to a statistical comparison between this group and CsA@F127DA/DA-PEG-GelMA group.

In order to further confirm whether the mechanism of hydrogel’s ability to restore cell viability was due to its internal antioxidant activity, which effectively inhibited cell apoptosis induced by H_2_O_2_, relevant studies were performed. ROS levels were determined in HCECs by DCFH-DA assay to quantify intracellular ROS levels [26]. ROS levels are an important marker of normal physiological cell function and oxidative cell damage caused by environmental factors [27,28]. Intracellular ROS levels were quantified by DCFH-DA fluorescence microscopy observation and DCFH-DA flow cytometry analysis. The results showed that ROS levels in HCECs soared significantly after exposure to H_2_O_2_. All three groups of hydrogels reduced cellular ROS level to a certain extent. However, it is noteworthy that CsA@F127DA/DA-PEG-GelMA was the most effective in clearing ROS. Interestingly, the ability of DA-PEG-GelMA to reduce ROS was superior to CsA@F127DA, which may be related to the fact that DA-PEG-GelMA was endowed with the antioxidant properties of DA (Fig. 4C&4D). The modification efficiency of DA has been verified by ABTS kit. DA at a concentration of 1–4 mg/mL exhibits excellent antioxidant properties, and its antioxidant performance is comparable to that of 1.5 mM vitamin E.

Additionally, immunofluorescence staining demonstrated that the hydrogels upregulated the expression levels of antioxidant markers, including catalase (CAT) and SOD1, in cells following H_2_O_2_ exposure (Figs. 4E&4F and S14). Cells were harvested for qRT-PCR to quantify the mRNA expression of these antioxidant markers, further assessing intracellular redox status. The qRT-PCR results for CAT and SOD1 expression aligned with the aforementioned findings (Figs. 4F and S15).

Chronic exposure of the ocular surface to adverse reactions can lead to the leakage of electrons from the mitochondrial respiratory chain, resulting in excessive production of ROS [29,30]. This alteration further affected the morphology and function of mitochondria, exacerbating oxidative stress damage to the ocular surface. Consequently, a vicious cycle is triggered, ultimately leading to tear film hyperosmolarity and ocular surface inflammation [8]. TOM20 serves as an indicator of the outer mitochondrial membrane, facilitating the transportation of cytoplasmically synthesized mitochondrial proteins across this membrane. Additionally, it plays a crucial role in sustaining the mitochondrial membrane potential and facilitating the transport of various substances. Cells were subjected to an oxidative stress model induced by H_2_O_2_. Compared with the CTRL group, the mitochondrial fragmentation in hydrogen peroxide was evident. The length to width ratio of mitochondria in H_2_O_2_ was reduced. This was consistent with the expected pattern of mitochondrial damage. However, the application of CsA@F127DA/DA-PEG-GelMA led to a more favorable rod-like morphology of intracellular mitochondria, indicating its protective role (Fig. S16).

In healthy cellular contexts, the mitochondrial inner membrane exhibits a negative transmembrane potential, in contrast to the positive potential of the outer membrane. Mito-Tracker is a cationic permeable dye that can penetrate the active mitochondrial membrane under the action of membrane potential and accumulate in the inner mitochondrial membrane. Intracellular mitochondria mostly clustered around the nucleus under H_2_O_2_ treatment. The application of CsA@F127DA/DA-PEG-GelMA dispersed the mitochondrial distribution (Fig. 4G&4J). Mitochondrial membrane potential (Δψm) is another major determinant and indicator of cellular mitochondrial function. Upon the initiation of apoptosis, the mitochondrial membrane potential diminishes, resulting in the release of TMRE into the cytoplasm and a notable decrease in the intensity of orange fluorescence within the mitochondria. By observing the changes of fluorescence color and mitochondrial membrane potential, it was shown that the intracellular TMRE fluorescence intensity in H_2_O_2_ group was decreased, and TMRE in HCECs was slightly improved after the administration of CsA@F127DA and DA-PEG-GelMA. TMRE in HCECs was significantly improved after treatment with CsA@F127DA/DA-PEG-GelMA, suggesting that the damaged mitochondrial membrane potential of HCECs was restored (Fig. 4H&4J).

The morphology of mitochondria could be observed more directly through transmission electron microscopy (TEM). Under TEM, healthy mitochondria presented closed sacs surrounded by double membranes with regular ridge arrangement [31]. When damaged by H_2_O_2_ oxidative stress, the permeability of mitochondrial membrane increases, the morphology becomes swollen, the matrix density decreases, and the mitochondrial ridge decreases or disappears. After the use of CsA@F127DA/DA-PEG-GelMA, mitochondrial morphology became more regular, the number of mitochondrial cristae increased, and the matrix density returned to normal. It is suggested that CsA@F127DA/DA-PEG-GelMA has the function of protecting mitochondrial morphology (Fig. 4I). Furthermore, alterations in the structure and function of mitochondria led to a reduction in their adenosine triphosphate (ATP)-generating capacity under H_2_O_2_ oxidative stress. Upon the application of CsA@F127DA/DA-PEG-GelMA, mitochondrial function was significantly enhanced, and there is a substantial increase in the amount of ATP produced (Fig. S17).

### 3.5 CsA@F127DA/DA-PEG-GelMA exerts antioxidant properties by protecting mitochondria under HS stress

High osmolarity is one of the central features of DE [32]. Therefore, 500 mOsm level of hyperosmotic medium was also leveraged for ROS generation (Fig. 5A). CCK8 assay results showed that compared with the HS group, all three groups of hydrogels could rescue cell viability to some extent (Fig. 5B). Under HS stress environment, a scratch test was conducted on HCECs. The results showed that CsA@F127DA/DA-PEG-GelMA exhibited a better ability to promote the growth and migration of cells (Fig. S18). In addition, CsA@F127DA/DA-PEG-GelMA showed a ROS scavenging effect consistent with HS stress under HS environment (Fig. 5C&5D). The antioxidant capacity of this hydrogel system was explored by inducing ROS generation in HCECs, while the expression level and immunofluorescence staining level of SOD2 were raised under HS oxidative stress with CsA@F127DA/DA-PEG-GelMA (Fig. 5E&5F). Besides, the mitochondrial length-to-width ratio in HCECs treated with CsA@F127DA/DA-PEG-GelMA was significantly improved compared to that in the HS group (Figs. 5G and S19). TMRE accumulated in normal mitochondria, decreases in hypertonic injury, and enhances post-CsA@F127DA/DA-PEG-GelMA application (Fig. 5H&5J). TEM also exhibited the CsA@F127DA/DA-PEG-GelMA’s morphological protective effect on mitochondria under hypertonic stress (Fig. 5I). In addition, after the application of CsA@F127DA/DA-PEG-GelMA, there was a substantial increase in the amount of ATP produced by mitochondria under HS oxidative stress (Fig. S20).

**Fig. 5:**
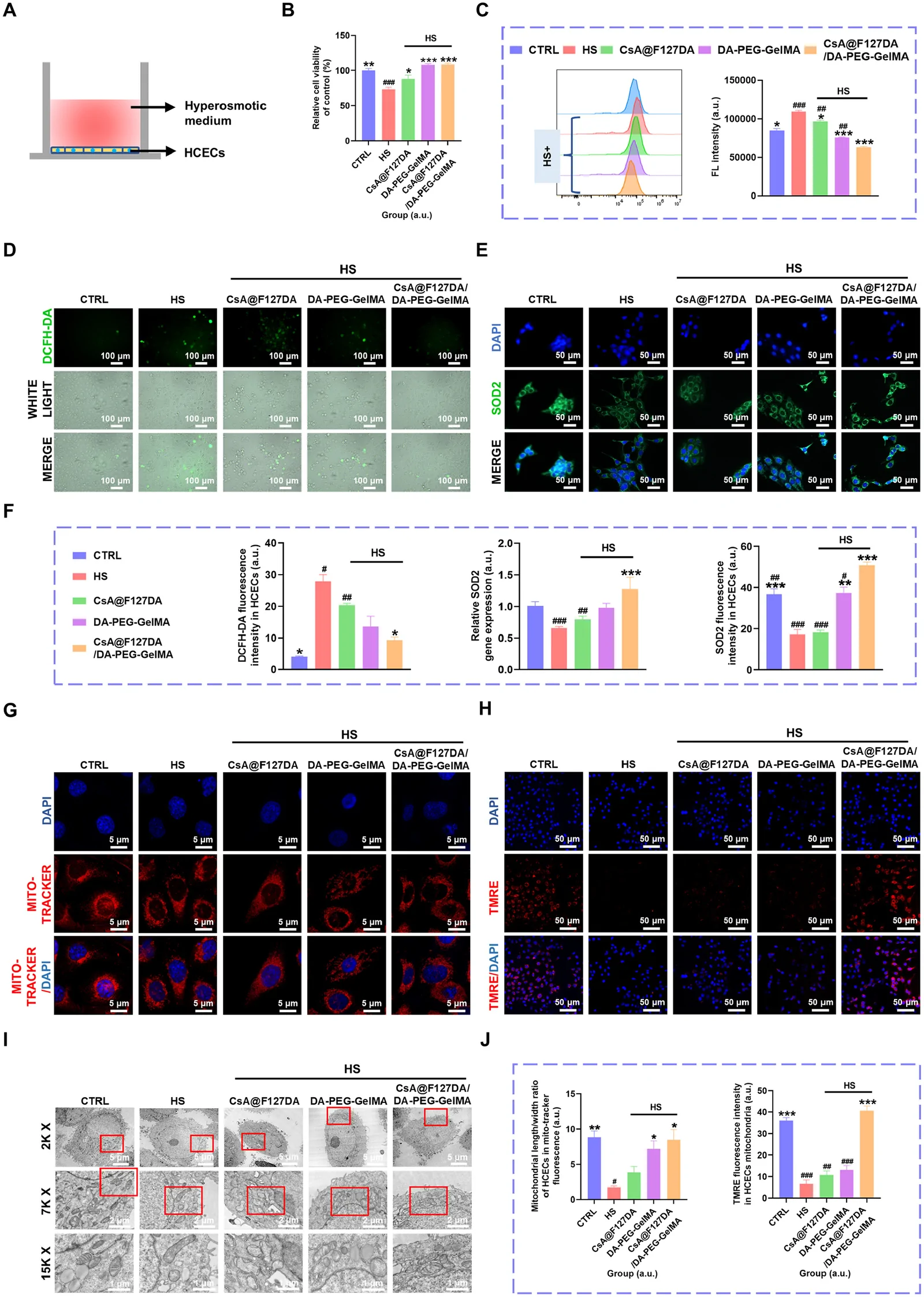
Antioxidant properties of CsA@F127DA/DA-PEG-GelMA under HS stress. (A) Schematic representation of hydrogel action on HCECs model of HS oxidative stress; (B) Cell viability of HCECs after HS exposure (*n* = 6 per group); (C) Quantification of ROS production levels in HCECs under HS oxidative stress by flow cytometry (*n* = 3 per group); (D) Representative images of ROS live cells staining and (E) SOD2 fluorescence staining under HS oxidative stress;. (F) Fluorescence intensity quantification of ROS live cells staining, and expression and fluorescence intensity quantification of SOD2 under HS oxidative stress (*n* = 3 to 6 per group); (G) Representative images of Mito-tracker staining, (H) TMRE staining and (I) mitochondrial TEM with different hydrogel treatments under HS oxidative stress; (J) Fluorescence intensity quantification of Mito-Tracker and TMRE staining with different hydrogel treatments under HS oxidative stress (*n* = 3 per group). Data are expressed as mean ± S.E.M., *P < 0.05, **P < 0.01, ***P < 0.001. ^#^*P* < 0.05, ^##^*P* < 0.01, ^###^*P* < 0.001.*Points to a statistical comparison between this group and HS group. ^#^Points to a statistical comparison between this group and CsA@F127DA/DA-PEG-GelMA group.

### 3.6 Anti-inflammatory properties of CsA@F127DA/DA-PEG-GelMA

Macrophages play an important role in the development of DE. Macrophages, serving as crucial phagocytic APCs within the immune system, become activated upon the initiation of DE. They play a pivotal role in secreting proinflammatory cytokines and chemokines, ultimately triggering subsequent adaptive immune responses [33,34]. Therefore, inhibiting the conversion of macrophage polarization to a pro-inflammatory phenotype is essential for ameliorating corneal epithelial damage and alleviating DE [12]. So as to explore the anti-inflammatory ability of the hydrogel system, we induced M1 type RAW 264.7 with LPS and IFN-γ (Fig. 6A). RAW 264.7 cells represent one of the most frequently utilized *in vitro* models in inflammation research. It is a kind of heterogeneous immune cells capable of being polarized into various phenotypes, enabling them to engage in both physiological and pathological responses of the body, influenced by different environments both *in vivo* and *in vitro*. RAW 264.7 cells differentiate to M1 type and produce pro-inflammatory factors through LPS and IFN-γ. qRT-PCR results showed that CsA@F127DA/DA-PEG-GelMA significantly reduced the expression of NOS2 and TNF-α in M1 type RAW 264.7 with statistically significant differences compared to the high expression of NOS2 and TNF-α in the MODEL group (Fig. 6B). CD86 and NOS2 are classic markers of RAW 264.7 type M1. Immunofluorescence staining also showed that CsA@F127DA/DA-PEG-GelMA decreased the expression of NOS2 (Fig. 6C) and CD86 (Fig. 6D). The expression of CD86 in RAW 264.7 cells with M1-type polarization was detected by flow cytometry. The results showed that the M1-type macrophage surface marker CD86 increased after LPS and IFN-γ stimulation of RAW 264.7 compared with the CTRL group, indicating successful M1-type polarization. All three hydrogels could reduce the transformation of RAW 264.7 into M1 proinflammatory phenotype. Among them, CsA@F127DA/DA-PEG-GelMA had the best effect on inhibiting the M1-type polarization of RAW 264.7. Interestingly, CsA@F127DA had a better inhibitory effect than DA-PEG-GelMA. This may be related to the fact that CsA@F127DA contains CsA, which has anti-inflammatory effects (Fig. 6E).

**Fig. 6:**
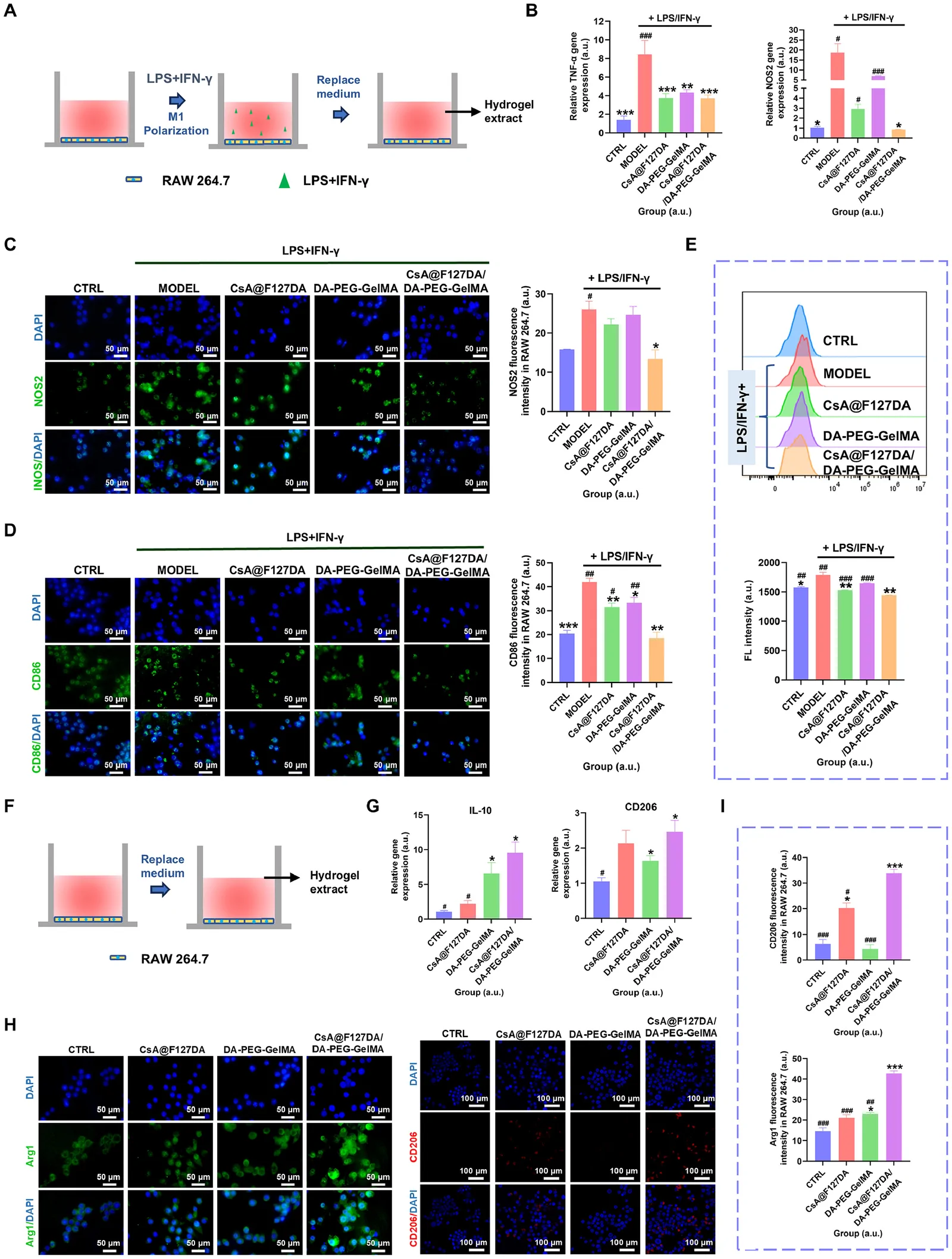
Anti-inflammatory properties of CsA@F127DA/DA-PEG-GelMA. (A) Schematic diagram of RAW 264.7 M1 type polarization model polarized by IFN-γ and LPS; (B) Expression of TNF-α and NOS2 in RAW 264.7 M1 type polarization model after different hydrogel effects (*n* = 6 per group); Representative images and fluorescence intensity quantification of (C) NOS2 and (D) CD86 in RAW 264.7 cells after RAW 264.7 M1 type polarization after different hydrogel effects (*n* = 3 per group); (E) Expression and quantification of CD86 in RAW 264.7 after M1-type polarization by flow cytometry (*n* = 3 to 6 per group); (F) Schematic diagram of RAW 264.7 M2 type polarization model polarized by hydrogels; (G) Expression of IL-10 and CD206 in RAW 264.7 M2 type polarization model after different hydrogel effects (*n* = 4 to 10 per group); (H) Representative images of Arg1 and CD206 in RAW 264.7 cells after different hydrogel effects; (I) Fluorescence intensity quantification of Arg1 and CD206 in RAW 264.7 cells after different hydrogel effects (*n* = 3 to 4 per group). Data are expressed as mean ± S.E.M., *P < 0.05, **P < 0.01, ***P < 0.001. ^#^*P* < 0.05, ^##^*P* < 0.01, ^###^*P* < 0.001. *Points to a statistical comparison between this group and MODEL group. ^#^Points to a statistical comparison between this group and CsA@F127DA/DA-PEG-GelMA group.

To investigate whether the hydrogel presents the function of inducing the transformation of RAW 264.7 cells to M2 type, a Transwell system loaded with hydrogel was utilized to simulate the action on RAW 264.7 (Fig. 6F). The expression levels of the M2 type markers IL10 and CD206 were measured by qRT-PCR. The results showed that CsA@F127DA/DA-PEG-GelMA increased the expression of IL-10 and CD206, which indicated that the hydrogel system might have the function of inducing the transformation of RAW 264.7 cells to M2 type (Fig. 6G). Immunofluorescence staining of RAW 264.7 cells for Arg1 and CD206 was performed. The fluorescence intensities of Arg1 and CD206 in RAW 264.7 were significantly higher than those in CsA@F127DA with DA-PEG-GelMA after the use of CsA@F127DA/DA-PEG-GelMA, suggesting that CsA@F127DA/DA-PEG-GelMA had a higher potential in inducing the transformation of RAW 264.7 cells to M2 type (Fig. 6H&6I).

Additionally, we investigated the anti-inflammatory properties of the hydrogel in HCECs. HS stress was established to simulate the DE. Inflammatory response in HCECs was significantly upregulated under HS stress. qRT-PCR results showed that after hydrogel treatment, IL-1β expression in the cells was significantly reduced, while the anti-inflammatory factor IL-13 was significantly upregulated (Fig. S21). Furthermore, WB analysis confirmed that the hydrogel could downregulate the expression of inflammatory factors IL-6 and IL-1β (Fig. S22). Under H_2_O_2_ oxidative stress, excessive accumulation of ROS not only induced oxidative stress in the cells but also promoted the release of inflammatory factors. Therefore, inflammatory factors in HCECs were assessed. Both qRT-PCR and WB results showed that hydrogel treatment significantly downregulated TNF-α levels in HCECs. Notably, the reduction of TNF-α levels in DA-PEG-GelMA was significantly higher than in CsA@F127DA, which may be attributed to group DA-PEG-GelMA’s superior ability to reduce ROS, thereby decreasing the activation of inflammatory factors at the source (Figs. S23 and S24).

### 3.7 Safety of CsA@F127DA/DA-PEG-GelMA in vivo

When applied to the ocular surface, the hydrogel system demands a thorough evaluation of its safety in mice, as the assessment has a direct bearing on subsequent experimental results. Such rigorous safety evaluation is imperative to guarantee that the eye drops effectively treat DE without eliciting any adverse effects on the eye. Furthermore, the duration of hydrogel adhesion on the ocular surface was investigated. Extended drug action on the ocular surface heightens the likelihood of localized chronic immune responses, compounded by the complexity of the hydrogel composition. Hence, ensuring a high level of safety and excellent biocompatibility on the ocular surface is paramount.

The ocular surface of mice was evaluated by slit lamp, *in vivo* confocal microscope and anterior segment OCT, and the fundus was evaluated by fundus photography and posterior segment OCT following administration of eye drops for 7 d to observe whether there were any side effects on the eyes (Fig. S25). After administration of CsA@F127DA, DA-PEG-GelMA and CsA@F127DA/DA-PEG-GelMA, the corneas of the mice showed transparency and no obvious sodium fluorescein staining (Fig. S26). The cornea structurally divides into five layers, including the epithelial layer, Bowman layer, stromal layer, Descemet layer and endothelial cell layer [35]. Corneal confocal microscopy showed that the corneal epithelial layer, stromal layer and endothelial layer in each group maintained normal morphology without obvious abnormal changes (Fig. S27). And the anterior segment OCT results indicated that there was no statistically significant difference in the corneal epithelial thickness between the treatment group and the CTRL group (Fig. S28). In addition, the posterior segment OCT and fundus photography results showed that the retinal layers were orderly arranged, and there were no significant changes in retinal blood vessels, and no obvious retinal degeneration, hemorrhage, leakage and other abnormal changes (Figs. S28 and S29). After treatment, the mice were euthanized, and their eyeballs were sectioned and subjected to H&E staining in order to examine the structural features of the cornea, conjunctiva and retina. Both the CTRL group and the treatment group present intact structure of the cornea, conjunctiva and retina, and no corneal defects or retinal detachment were observed (Fig. S30). All the group, CsA@F127DA, DA-PEG-GelMA and CsA@F127DA/DA-PEG-GelMA displayed good safety and biocompatibility in the eyes, suggesting the possibility of their clinical application in ophthalmology.

To evaluate the mid- to long-term safety of this formulation and 405 nm blue light irradiation, we conducted a 3-month repeated-dose safety study. After 3 months, ocular examinations were performed, including slit-lamp microscopy (Fig. S31), corneal confocal microscopy (Fig. S32), anterior segment OCT (Fig. S33), and fundus photography (Fig. S34). In addition, H&E staining was conducted on the eyes (Fig. S35) and main organs – heart, liver and kidney (Fig. S36). No significant formulation-related toxicological changes were observed. Importantly, the present 3-month safety evaluation was limited to gross morphology and lacked functional retinal assessments (ERG) or quantitative subbasal nerve plexus analysis. Given that LAP-induced free radicals may affect neural tissues, we cannot rule out the possibility of delayed phototoxic sequelae. Therefore, while these early results are encouraging, they should not be overinterpreted as conclusive proof of safety. Long-term studies incorporating ERG and corneal nerve density metrics are critically needed prior to considering human use.

### 3.8 CsA@F127DA/DA-PEG-GelMA alleviates DE

To gain a more profound understanding of the therapeutic effects of the hydrogel on DE, we established a mouse model of the condition by administering BAC twice daily for 7 d consecutivly [36] (Fig. 7A). CsA@F127DA, DA-PEG-GelMA, CsA@F127DA/DA-PEG-GelMA, F127DA/GelMA and Restasis® were additionally used in the treatment groups (Fig. 7B). Corneal epithelial sodium fluorescein staining and tear production measurements were pivotal indicators for assessing DE. Moreover, tear deficiency can disrupt the epithelial homeostasis, thereby intensifying the symptoms related to DE. Tear secretion results showed that all the groups of hydrogels increased tear production, with CsA@F127DA/DA-PEG-GelMA being the most effective, which implies that this hydrogel has a better DE treatment effect (Fig. 7C). Quantification of corneal epithelial sodium fluorescein staining by slit-lamp photography revealed that all the groups of hydrogels significantly reduced sodium fluorescein staining of the ocular surface compared to the MODEL group. After the application of CsA@F127DA, DA-PEG-GelMA, F127DA/GelMA and Restasis®, while the ocular surface sodium fluorescein staining showed marked improvement, a small area of punctate staining persisted. However, the corneal sodium fluorescein staining intensity after using the combination of CsA@F127DA/DA-PEG-GelMA was closer to that of the normal group. Notably, the fluorescein staining outcomes observed in mouse ocular tissues subsequent to the administration of CsA@F127DA eye drop exhibited a superior efficacy compared to that of CsA application alone. This enhanced performance could be attributed to the extended ocular retention time of CsA@F127DA, which facilitates the sustained maintenance of drug concentration over an elongated duration (Fig. 7D&7E).

**Fig. 7:**
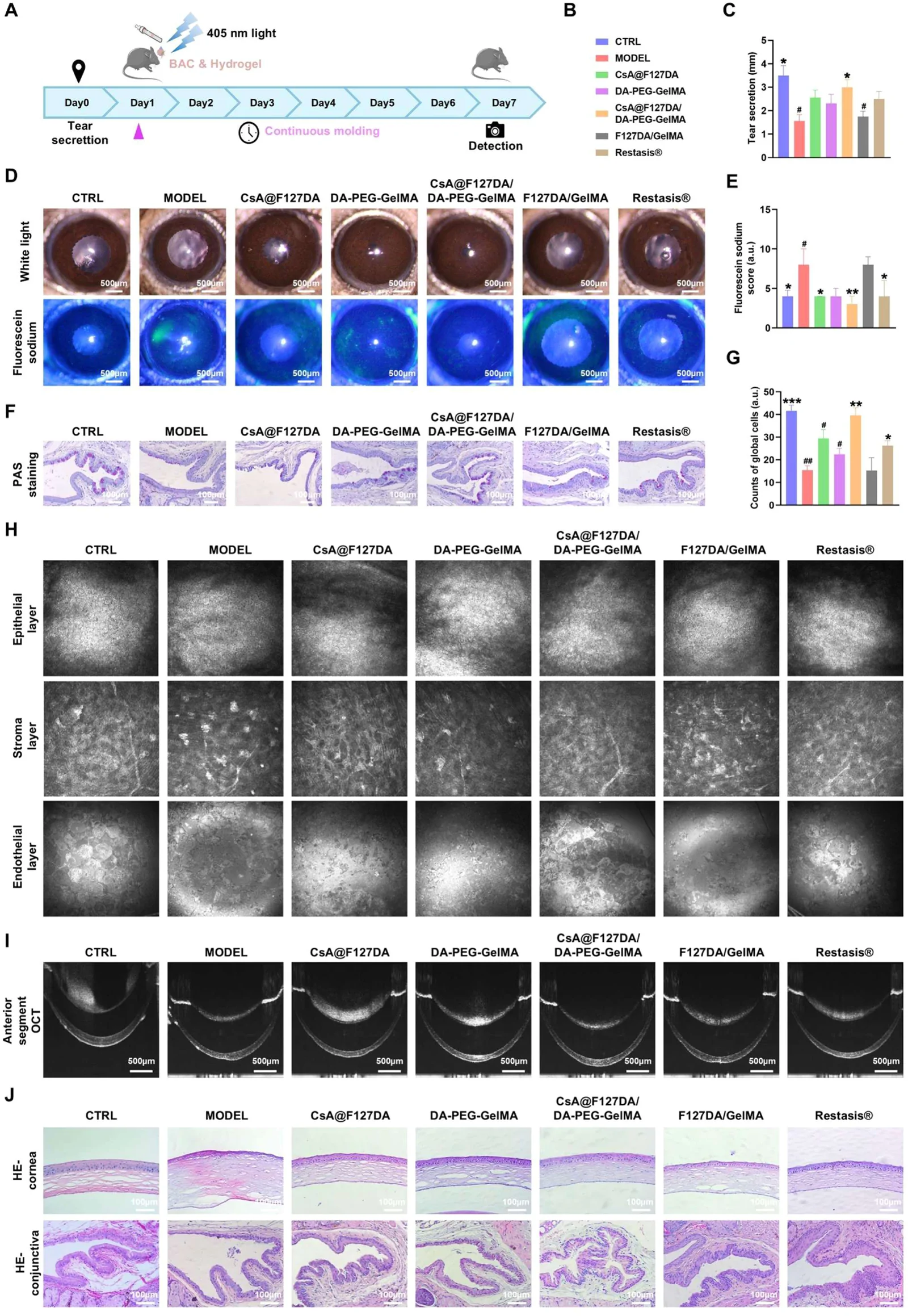
CsA@F127DA/DA-PEG-GelMA alleviates DE. (A) DE mice model administration timeline; (B) Grouping of DE experiment; (C) Statistical analysis of tear secretion (*n* = 8 per group); (D) Representative corneal images by corneal slit-lamp; (E) Statistical analysis of corneal fluorescence staining under slit lamp and evaluated scores quantification (*n* = 7 per group); (F) Representative images of goblet cells PAS staining; (G) Statistical analysis of goblet cells PAS staining (*n* = 5 to 8 per group); (H) Representative images of *in vivo* corneal laser confocal microscope images; (I) Representative OCT images of the anterior segment (the red arrow indicates the defects of corneal epithelium); (J) Representative images of H&E staining of corneal sections and conjunctiva sections; Data in E are presented as median ± IQR, while other data are expressed as mean ± S.E.M. *P < 0.05, **P < 0.01, ***P < 0.001. ^#^*P* < 0.05, ^##^*P* < 0.01. *Points to a statistical comparison between this group and MODEL group. ^#^Points to a statistical comparison between this group and CsA@F127DA/DA-PEG-GelMA group.

The tear fluid comprises three layers: the aqueous layer, the lipid layer, and the mucin layer [37]. Mucin, an essential component of tear fluid, plays a pivotal role in enhancing the stability of the tear film, prolonging tear film breakup time, and minimizing the ocular surface’s direct exposure to the complex and dynamic external environment [38]. Conjunctival goblet cells are the primary source of mucins and their count serves as a crucial indicator of DE severity. PAS staining of conjunctival goblet cells in the MODEL group revealed a substantial loss of goblet cells and a wrinkled morphology among the remaining cells, compared to the normal group. Notably, CsA@F127DA/DA-PEG-GelMA exhibited the most effective protective effect on goblet cells, suggesting its superiority in improving DE symptoms (Fig. 7F&7G).

Changes in the cell structure and number of corneal layers could be observed by *in vivo* confocal microscope. The stroma layer of MODEL group showed obvious inflammatory infiltration and increased epithelial cell loss. After using CsA@F127DA/DA-PEG-GelMA, the infiltration of fine inflammatory cells in the stroma of mice cornea was reduced, and the number and morphology of epithelial cells were greatly improved (Fig. 7H). The anterior segment OCT can provide insights into the thickness and smoothness of the cornea. After BAC administration, mice exhibited corneal epithelial defects and a decrease in smoothness. The combined use of CsA@F127DA with DA-PEG-GelMA showed some improvement in corneal defects. However, compared to other groups, the use of CsA@F127DA/DA-PEG-GelMA offered the most significant protection to the corneal epithelial barrier function, reducing edema and inflammatory infiltration. The corneal thickness appeared smoother and more uniform, suggesting that CsA@F127DA/DA-PEG-GelMA had the optimal effect in alleviating dry eye (Figs. 7I and S37).

It should be noted that OCT and H&E staining reflect different aspects of corneal changes, with OCT assessing *in vivo* corneal morphology and H&E revealing *ex vivo* tissue architecture. Therefore, differences between these measurements may result from methodological variations. In order to further evaluate the effect of hydrogel in improving DE and to explore the histological changes of corneal epithelium and conjunctival epithelium, we performed chemical staining. By H&E staining of the corneal conjunctiva, results showed that the corneal epithelium in the model group was disorganized, and the corneal epithelium appeared to be obviously detached. In the meantime, the cornea exhibited edema, accompanied by an elevated presence of vacuoles within the stroma layer. The morphological and structural changes observed in the cornea are consistent with previous reports on DE cases. In contrast, the corneal epithelial morphology remained intact in the treatment groups, particularly in the CsA@F127DA/DA-PEG-GelMA group, which exhibited reduced inflammatory infiltration and absence of corneal edema. Furthermore, the treatment group demonstrated a significant improvement in conjunctival goblet cell retention and atrophy, in stark contrast to the MODEL group, which showed sparse conjunctival goblet cell expression (Figs. 7J and S37). In summary, the results indicate that the hydrogel enhances corneal epithelial barrier function and alleviates conjunctival goblet cell loss, thereby hinting at its promising potential as a therapeutic option for clinical DE.

#### 3.8.1 CsA@F127DA/DA-PEG-GelMA maintains corneal redox balance

The aforementioned results suggest that CsA@F127DA/DA-PEG-GelMA holds promise for improving dry eye symptoms. To delve deeper into the specific mechanism by which this photocurable hydrogel alleviates DE symptoms, we employed immunofluorescence staining and qPCR to assess the levels of anti-oxidative stress markers and inflammatory factors in the cornea and conjunctiva. The qPCR findings indicated that CsA@F127DA, DA-PEG-GelMA and CsA@F127DA/DA-PEG-GelMA all effectively reduced the expression of MMP3 in the cornea. Besides, qPCR results suggested that DA-PEG-GelMA had a better ability to increase the anti-oxidative stress indicators HO-1, SOD1, CAT and SOD2 than CsA@F127DA, which might be associated with its DA modification and increased antioxidant capacity (Figs. 8A and S38). The excellent improved corneal barrier function and antioxidant properties of CsA@F127DA/DA-PEG-GelMA were further demonstrated by fluorescent staining of the cornea for MMP3, HO-1, SOD1 and CAT (Fig. 8B&8 C). Additionally, in addition, GSEA suggested that CsA could inhibit apoptosis of corneal epithelial cells and reduce their inflammatory response (Fig. S39). Therefore, we conducted TUNEL staining on corneas and found that CsA@F127DA/DA-PEG-GelMA significantly inhibited cell apoptosis in corneal epithelial cells (Fig. S40).

**Fig. 8:**
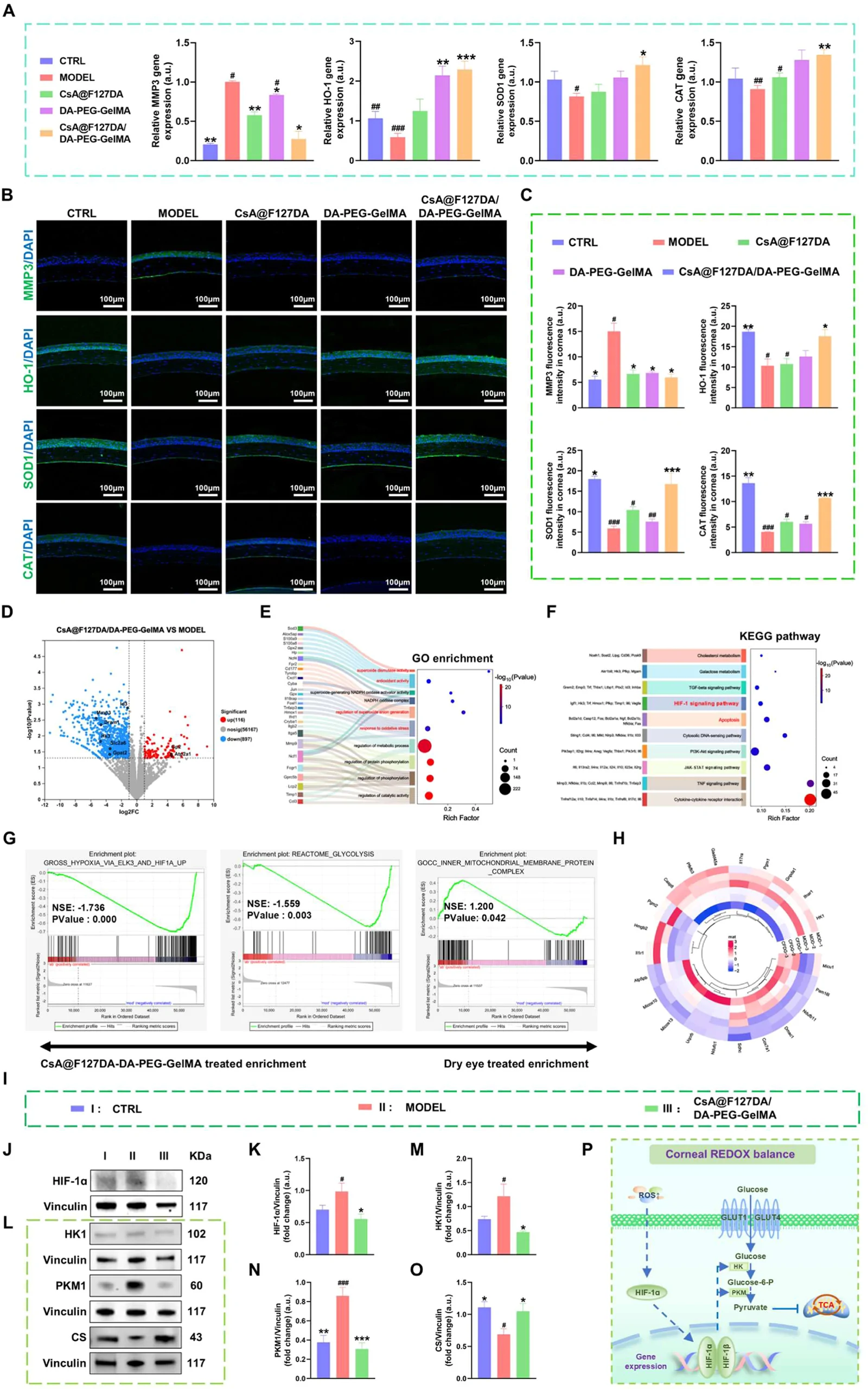
CsA@F127DA/DA-PEG-GelMA maintains corneal redox balance. (A) Expression (*n* = 4 to 6 per group), (B) representative images and (C) fluorescence intensity quantification (*n* = 3 to 4 per group) of corneal MMP3, HO-1, SOD1 and CAT under DE; (D) Volcano plot of differentially expressed corneal genes; (E) GO functional enrichment and gene relationships; (F) KEGG pathway enrichment and gene relationships; (G) GSEA of HIF-1 signaling, glycolysis, and mitochondrial membrane protein complex; (H) Heatmaps of genes related to HIF-1 signaling, glycolysis, and mitochondrial function.; (I) Experimental groups: CTRL, MODEL, CsA@F127DA, DA-PEG-GelMA and CsA@F127DA/DA-PEG-GelMA; (J) Corneal HIF-1α protein expression; (K) HIF-1α quantification (*n* = 4 per group); (L) Corneal HK1, PKM1 and CS protein expression; (M) Protein expression of HK1 (*n* = 3 per group), (N) PKM1 (*n* = 6 per group), (O) and CS (*n* = 5 per group); (P) Schematic of HIF-1 signaling. Data are expressed as mean ± S.E.M. *P<0.05, **P<0.01, ***P<0.001. ^#^*^P^*<0.05, ^##^*P*<0.01, ^###^*P*<0.001. *Points to a statistical comparison between this group and MODEL group. #Points to a statistical comparison between this group and CsA@F127DA/DA-PEG-GelMA group.

To further explore the specific mechanism by which CsA@F127DA/DA-PEG-GelMA enhanced corneal barrier function, we performed RNA sequencing analysis on the CsA@F127DA/DA-PEG-GelMA-treated cornea compared with the untreated model group cornea. The results of differential gene analysis revealed significant gene expression differences between the two groups, including 116 up-regulated genes and 897 down-regulated genes. Notably, in the CsA@F127DA/DA-PEG-GelMA treatment group, the expression of genes such as MMP3 and HK3 was significantly down-regulated, while the expression of ATP-related genes was significantly up-regulated (Fig. 8D). This finding suggests that CsA@F127DA/DA-PEG-GelMA may alleviate the oxidative stress state of the cornea by regulating the energy metabolism of the cells, and then improve the barrier function of the cornea. To further explore the main functions and pathways involved in CsA@F127DA/DA-PEG-GelMA in the treatment of DE, pathway enrichment analysis was further performed. The results of mulberry diagram showed that GO and KEGG analysis suggested that the enriched genes were mainly concentrated in the functions related to antioxidant activity, oxidative stress response and phosphorylation. This suggests that CsA@F127DA/DA-PEG-GelMA may improve corneal barrier function and relieve DE symptoms by regulating HIF-1αsignaling pathway, reducing mitochondrial oxidative stress damage (Fig. 8E&8F). GSEA analysis further verified the expression trend of HIF-1 signaling pathway (Fig. 8G). The heat map further showed the differential expression of targets in HIF-1 signaling pathway, glycolysis and mitochondrial membrane protein complex between CsA@F127DA/DA-PEG-GelMA group and MODEL group (Fig. 8H).

Under DE conditions, inflammation and oxidative stress mutually reinforce each other, forming a self-amplifying pathological loop; meanwhile, mitochondrial dysfunction can lead to elevated levels of ROS. Excessive production of ROS disrupts mitochondrial redox homeostasis, thereby aggravating oxidative stress–associated damage and accelerating the vicious cycle of DE [39]. At the molecular level, elevated ROS can inhibit prolyl hydroxylase (PHD) activity, thereby reducing HIF-1α hydroxylation and degradation and promoting its stabilization. In DE, where tear film instability coexists with local hypoxia in the corneal epithelium, the HIF-1 signaling pathway can be markedly activated [40,41]. Sustained activation of HIF-1 further drives the expression of key glycolytic enzymes HK1 and PKM1 and suppresses the expression of the mitochondrial respiration–related gene CS, thereby reinforcing metabolic reprogramming and exacerbating redox imbalance, ultimately perturbing mitochondrial redox homeostasis [[42], [43], [44]]. To further explore the regulatory effects of CsA@F127DA/DA-PEG-GelMA on mitochondrial glycolysis via the HIF-1α signaling pathway, we detected related protein expression in corneas under different treatments by WB (Fig. 8I). The results showed that the expression levels of HIF-1α decreased after the administration of CsA@F127DA/DA-PEG-GelMA (Fig. 8, Fig. 8K). HK1 and PKM1 are key enzymes in glycolysis. CS is key enzyme in the tricarboxylic acid (TCA) cycle. The results showed that in the cornea of the DE model group, the expression levels of HK1 and PKM1 increased, the expression level of CS decreased, while after the administration of CsA@F127DA/DA-PEG-GelMA, the expression levels of HK1 and PKM1 decreased, the expression level of CS increased (Fig. 8L–8O). The results showed that CsA@F127DA/DA-PEG-GelMA effectively reduced mitochondrial oxidative stress damage by inhibiting the HIF-1 signaling pathway, enhancing the regulation of cellular energy metabolism and reducing the occurrence of cell glycolysis This finding provides a new perspective for our understanding of how CsA@F127DA/DA-PEG-GelMA improves corneal barrier function and provides a potential rationale for its use in the treatment of DE (Fig. 8P).

### 3.9 CsA@F127DA/DA-PEG-GelMA maintains the immune homeostasis of the conjunctiva

The immune homeostasis of the conjunctiva is essential for maintaining the balance of the ocular surface microenvironment. Conjunctival qRT-PCR results showed that CsA@F127DA/DA-PEG-GelMA reduced conjunctival IL-1β, IL-6 and IFN-γ expression, increased IL-13 expression. The gene expression level of MUC5AC was also upregulated by CsA@F127DA/DA-PEG-GelMA (Fig. 9A). MUC5AC serves as a marker for conjunctival mucins. Conjunctival immunofluorescence staining revealed that all three hydrogel groups had the capacity to enhance conjunctival MUC5AC expression compared to the MODEL group. Notably, CsA@F127DA/DA-PEG-GelMA demonstrated the highest efficacy (Fig. 9B). This also correlated with the mice exhibiting a greater ability to mitigate the loss of conjunctival goblet cells after the application of CsA@F127DA/DA-PEG-GelMA, suggesting its significant potential in ophthalmological clinical applications. Furthermore, immunofluorescence analysis elegantly demonstrated suppressive influence of CsA@F127DA/DA-PEG-GelMA on inflammation. Specifically, IL-1β expression was prominently elevated in the MODEL group, whereas it was distinctly decreased in the CsA@F127DA/DA-PEG-GelMA group, underscoring its anti-inflammatory potential (Fig. 9C). DA-PEG-GelMA demonstrated an anti-inflammatory ability comparable to that of CsA@F127DA. This may stem from its vicious circle of inhibiting DE in terms of antioxidant, which in turn ameliorates the inflammatory infiltration of the conjunctiva. However, it is noteworthy that CsA@F127DA/DA-PEG-GelMA exhibited superior performance in enhancing corneal barrier function, bolstering corneal anti-oxidative stress capabilities, and inhibiting conjunctival inflammatory infiltration. This superiority may be attributed to its combined anti-inflammatory properties derived from CsA@F127DA and anti-oxidative stress properties from DA-PEG-GelMA, which effectively disrupt the vicious cycle of inflammation and oxidative stress in the progression of DE. This indicates that CsA@F127DA/DA-PEG-GelMA holds greater potential in ameliorating DE compared to CsA@F127DA and DA-PEG-GelMA alone.

**Fig. 9:**
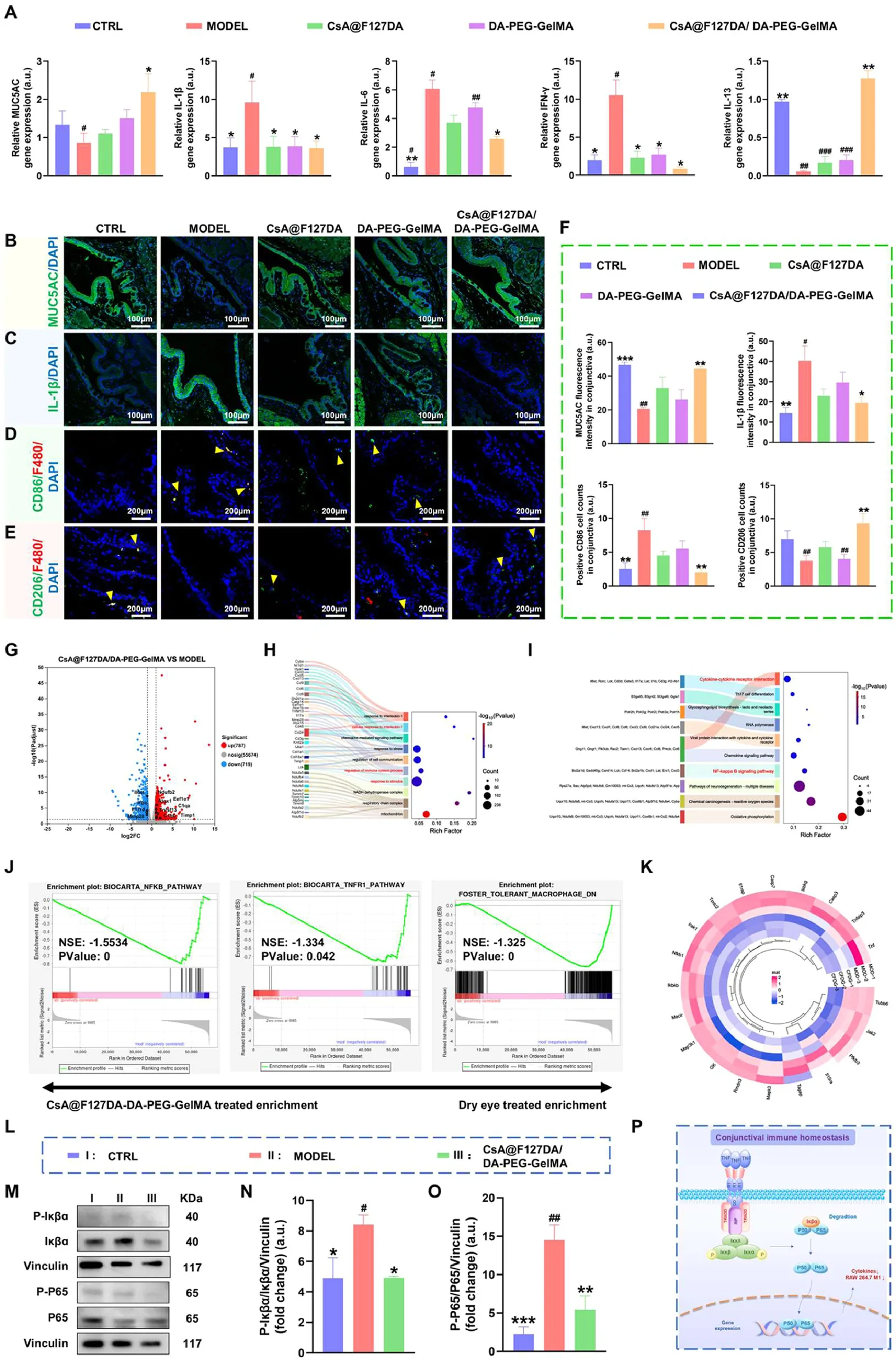
CsA@F127DA/DA-PEG-GelMA maintains conjunctival immune homeostasis. (A) Relative expression of conjunctival anti-inflammatory and antioxidant gene under DE (*n* = 3 to 12 per group); Representative images of conjunctival (B) MUC5AC, (C) IL-1β, (D) CD86 and (E) CD206 under DE; (F) Fluorescence intensity quantification of MUC5AC and IL-1β and counts of CD86 and CD206-positive cells (*n* = 4 to 5 per group); (G) Volcano plot of differentially expressed conjunctival genes; (H) GO functional enrichment and gene relationships; (I) KEGG pathway enrichment and gene relationships; (J) GSEA of NF-κB signaling, TNF-α signaling, and macrophage-related targets; (K) Heatmaps of genes associated with NF-κB signaling, TNF-α signaling, and macrophage-related targets; (L) Experimental groups: CTRL, MODEL, CsA@F127DA, DA-PEG-GelMA, and CsA@F127DA/DA-PEG-GelMA; (M) Conjunctival protein expression of P-Ikβα, Ikβα, p65 and p-p65; (N) Quantification of P-Ikβα (*n* = 3 per group) and (O) p-p65 (*n* = 4 per group), respectively; (P) Schematic of NF-κB signaling. Data are expressed as mean ± S.E.M. *P<0.05, **P<0.01, ***P<0.001. ^#^*P*<0.05, ^##^*P*<0.01, ^###^*P*<0.001. *Points to a statistical comparison between this group and MODEL group. #Points to a statistical comparison between this group and CsA@F127DA/DA-PEG-GelMA group.

As the main component of immune cells, macrophages play a major role in the immunomodulatory process of DE [45]. CsA could effectively acts on macrophages [46]. To further explore the association between CsA@F127DA/DA-PEG-GelMA and macrophages, we performed co-staining of F480 and CD86 or CD206. The results showed that CD86 was decreased and CD206 was increased in the conjunctiva after the administration of CsA@F127DA/DA-PEG-GelMA, indicating that CsA@F127DA/DA-PEG-GelMA could promote the polarization of macrophages to M2 type and inhibit their polarization to M1 type (Figs. 9D&9E and S41). The specific impact on macrophages observed may be attributed to the unique interplay between CsA and macrophages. The above staining was statistically significant (Fig. 9F).

To further explore the mechanism of improving conjunctival inflammation and alleviating DE, we used conjunctiva of the CsA@F127DA/DA-PEG-GelMA treated group and the MODEL group for transcriptome analysis. The results showed that there were significant differences between the two groups, with 787 genes up-regulated and 719 genes down-regulated. Notably, both the matrix metalloproteinase family and the interleukin family were significantly down-regulated genes (Fig. 9G). The mulberry plot demonstrates the major functional targets and signaling pathway of CsA@F127DA/DA-PEG-GelMA for DE treatment. The mulberry plot shows the main functional targets and signaling pathways of CsA@F127DA/DA-PEG-GelMA for DE treatment. These results suggest that the interaction between CsA@F127DA/DA-PEG-GelMA and IL-1 may be related to the chemokine family (Ccl). This process, in turn, regulates the function of the NF-κB signaling pathway and reduces the interaction between conjunctival epithelial cells and immune cells, thereby inhibiting the formation and development of an inflammatory immune microenvironment driven by immune cells (Fig. 9H&9I). GSEA further demonstrated the inhibitory effect of CsA@F127DA/DA-PEG-GelMA on inflammation and revealed its role in promoting macrophage polarization to M2 type (Fig. 9J). The heat map further visually reveals the close association between the internal key targets identified in the GSEA analysis and the regulation of CsA@F127DA/DA-PEG-GelMA (Fig. 9K). In addition, GSEA suggested that CsA@F127DA/DA-PEG-GelMA could improve conjunctival apoptosis, reduce the production of cell-related death signals, and enhance the oxidative phosphorylation process of the conjunctiva (Figs. S42–S44).

To further explore the specifics of the NF-κB signaling pathway regulated by CsA@F127DA/DA-PEG-GelMA, WB was performed on conjunctiva under different treatments (Fig. 9L). The results showed that the expression levels of P-Iκβα and P-P65 in the conjunctiva of the DE model group increased, while the expression levels of P-Iκβα and P-P65 in the conjunctiva decreased after the administration of CsA@F127DA/DA-PEG-GelMA (Fig. 9M–9O). This indicates that CsA@F127DA/DA-PEG-GelMA inhibits the activation of the NF-κB signaling pathway (Fig. 9P). The regulation of the NF-κB signaling pathway in RAW 264.7 was also assessed. The results demonstrated that, the expression levels of P-Iκβα and P-P65 in RAW 264.7 under M1 type polarization were significantly reduced under CsA@F127DA/DA-PEG-GelMA treatment. This further confirms the inhibitory effect of the hydrogel on the NF-κB signaling pathway (Fig. S45).

### 3.10 CsA@F127DA/DA-PEG-GelMA improves lipid secretion of meibomian gland

The structural and functional integrity of the meibomian gland is crucial for maintaining a healthy ocular surface. The tear film, composed of an aqueous layer, lipid layer and mucin layer, relies on the meibomian glands to secrete lipids, which are essential for tear film stability. When meibomian gland dysfunction occurs, qualitative and quantitative abnormalities in the lipid layer of the tear film can be observed. Direct microscopic observation (Fig. S46) and H&E staining (Fig. S47) of the meibomian gland morphology revealed that CsA@F127DA/DA-PEG-GelMA use led to improved gland atrophy compared to the MODEL group. Oil red staining of lipids in the meibomian glands provided a clearer view of lipid secretion. Notably, significant oil red staining in the MODEL group suggested potential blockages in the meibomian gland openings, impeding proper lipid secretion (Fig. S48). Similarly, LipidTox staining confirmed these findings (Fig. S49).

## 4 Conclusion

In summary, this study successfully synthesized CsA@F127DA/DA-PEG-GelMA and elucidated its dual pharmacological activities to improve corneal epithelial glucose metabolism and maintain mitochondrial redox homeostasis by inhibiting HIF-1 signaling pathway, and to improve conjunctival inflammatory infiltration by inhibiting NF-κB signaling pathway. These interconnected regulatory axes orchestrate a self-perpetuating reparative cycle that restores ocular surface homeostasis through multi-dimensional mechanisms, alleviating symptoms in DE model mice. This study not only provides new insights into the pathological mechanism of DE, but also fills a gap in the application of photocurable hydrogels as drug delivery systems for DE, offers a theoretical reference for their use in ophthalmology, and lays a foundation for their potential use in treating other inflammatory diseases or mitochondrial homeostasis disorders.

## CRediT authorship contribution statement

**Qinghe Zhang:** Writing – original draft, Visualization, Validation, Software, Methodology, Investigation, Data curation, Conceptualization. **Ke Yan:** Writing – original draft, Visualization, Validation, Software, Methodology, Investigation, Formal analysis, Data curation. **Panqin Ma:** Visualization, Validation, Methodology, Investigation, Data curation, Conceptualization. **Weilu He:** Methodology, Data curation. **Minyan Wei:** Methodology, Data curation. **Qiuping Liu:** Writing – review & editing, Supervision, Resources, Project administration, Funding acquisition. **Yi Han:** Writing – review & editing, Writing – original draft, Supervision, Resources, Project administration, Funding acquisition, Conceptualization. **Zuguo Liu:** Writing – review & editing, Supervision, Project administration, Funding acquisition, Conceptualization.

## Conflicts of interest

The authors declare that there is no conflicts of interest.

## Appendix Supplementary materials

Image, application 1

Image, application 2

Image, application 3

Image, application 4

Image, application 5

## References

[1] Craig J.P., Nichols K.K., Akpek E.K., Caffery B., Dua H.S., Joo C.K. (2017). TFOS DEWS II definition and classification report. Ocul Surf.

[2] Stapleton F., Alves M., Bunya V.Y., Jalbert I., Lekhanont K., Malet F. (2017). TFOS DEWS II epidemiology report. Ocul Surf.

[3] Zong Y., Yang Y., Zhao J., Li L., Luo D., Hu J. (2023). Characterisation of macrophage infiltration and polarisation based on integrated transcriptomic and histological analyses in primary Sjögren’s syndrome. Front Immunol.

[4] Arras M., Strasser R., Mohri M., Doll R., Eckert P., Schaper W. (1998). Tumor necrosis factor-alpha is expressed by monocytes/macrophages following cardiac microembolization and is antagonized by cyclosporine. Basic Res Cardiol.

[5] Matsubara Y., Kiwan G., Liu J., Gonzalez L., Langford J., Gao M. (2021). Endothelial SIRT6 limits vascular inflammation and atherosclerosis by suppressing NF-κB signaling. Arterioscler Thromb Vasc Biol.

[6] Bai X., Yang W., Li H., Zhao Y., Fan W., Zhang H. (2022). Cyclosporine A regulates influenza A virus-induced macrophages polarization and inflammatory responses by targeting cyclophilin A. Front Immunol.

[7] Yang D., Han Y., Wang Y., Pan Y., Zheng L., Liu Z. (2023). Injectable reactive oxygen species-scavenging hydrogel regulates macrophage polarization for treating dry eye disease. Chem Eng J.

[8] Xia Y., Zhang Y., Du Y., Wang Z., Cheng L., Du Z. (2024). Mitochondria-targeted hydrogel ameliorates ocular inflammation via modulating macrophage metabolism and M2 polarization. J Nanobiotechnol.

[9] Spinelli J.B., Haigis M.C. (2018). The multifaceted contributions of mitochondria to cellular metabolism. Nat Cell Biol.

[10] Willems PHGM, Rossignol R., Dieteren C.E.J., Murphy M.P., Koopman W.J.H. (2015). Redox homeostasis and mitochondrial dynamics. Cell Metab.

[11] Dunn J.D., Alvarez L.A., Zhang X., Soldati T. (2015). Reactive oxygen species and mitochondria: a nexus of cellular homeostasis. Redox Biol.

[12] Li S., Lu Z., Huang Y., Wang Y., Jin Q., Shentu X. (2022). ROS-scavenging hydrogel to promote healing of bacteria infected diabetic wounds. Adv Sci.

[13] Nagai N., Otake H. (2022). Ocular drug delivery and barriers: an updated review. Adv Drug Deliv Rev.

[14] Gote V., Sikder S., Sicotte J., Pal D. (2019). Ocular drug delivery: present challenges and future strategies. J Pharmacol Exp Ther.

[15] Zhang Q., Yan K., Zheng X., Liu Q., Han Y., Liu Z. (2024). Research progress of photo-crosslink hydrogels in ophthalmology: a comprehensive review focus on the applications. Mater Today Bio.

[16] Eshel-Green T., Bianco-Peled H. (2016). Mucoadhesive acrylated block copolymers micelles for the delivery of hydrophobic drugs. Colloids Surf B Biointerfaces.

[17] Yue K., Trujillo-de Santiago G., Alvarez M.M., Tamayol A., Annabi N., Khademhosseini A. (2015). Synthesis, properties, and biomedical applications of gelatin methacryloyl (GelMA) hydrogels. Biomaterials.

[18] Kurian A.G., Singh R.K., Patel K.D., Lee J.H., Kim H.W. (2022). Multifunctional hydrogel systems for immunomodulation and tissue regeneration. Bioact Mater.

[19] Li M., Wei R., Liu C., Fang H., Yang W., Wang Y. (2023). A “T.E.S.T.” hydrogel bioadhesive assisted by corneal cross-linking for *in situ* sutureless corneal repair. Bioact Mater.

[20] Kang H., Han Y., Jin M., Zheng L., Liu Z., Xue Y. (2023). Decellularized squid mantle scaffolds as tissue-engineered corneal stroma for promoting corneal regeneration. Bioeng Trans Med.

[21] Hoffman A.S. (2002). Hydrogels for biomedical applications. Adv Drug Deliv Rev.

[22] Han L., Lu X., Liu K., Wang K., Fang L., Weng L.T. (2017). Mussel-inspired adhesive and tough hydrogel based on nanoclay confined dopamine polymerization. ACS Nano.

[23] Perikamana S.K.M., Lee J., Lee Y.B., Shin Y.M., Lee E.J., Mikos A.G. (2015). Surface modifications of biomaterials to modulate macrophage polarization for tissue regeneration. Adv Healthc Mater.

[24] Seen S., Tong L. (2018). Dry eye disease and oxidative stress. Acta Ophthalmol.

[25] Zhou T., Zong R., Zhang Z., Zhu C., Pan F., Xiao X. (2012). SERPINA3K protects against oxidative stress via modulating ROS generation/degradation and KEAP1-NRF2 pathway in the corneal epithelium. Invest Ophthalmol Vis Sci.

[26] Ahmad A., Ahsan H. (2020). Biomarkers of inflammation and oxidative stress in ophthalmic disorders. J Immunoassay Immunochem.

[27] Brieger K., Schiavone S., Miller F.J., Krause K.H. (2012). Reactive oxygen species: from health to disease. Swiss Med Wkly.

[28] Cardoso M.A., Gonçalves H.M.R., Davis F. (2023). Reactive oxygen species in biological media are they friend or foe? Major *in vivo* and *in vitro* sensing challenges. Talanta.

[29] Mazat J.P., Devin A., Ransac S. (2020). Mitochondrial ROS metabolism: regulation of ROS production, ROS oxidation and ROS emission. Cell Mol Life Sci.

[30] Guan S., Zhao L., Peng R. (2022). Mitochondrial respiratory chain supercomplexes: from structure to function. Int J Mol Sci.

[31] Sun M.G., Williams J., Munoz-Pinedo C., Perkins G.A., Brown J.M., Ellisman M.H. (2007). Correlated three-dimensional light and electron microscopy reveals transformation of mitochondria during apoptosis. Nat Cell Biol.

[32] Bron A.J., De Paiva C.S., Chauhan S.K., Bonini S., Gabison E.E., Jain S. (2017). TFOS DEWS II pathophysiology report. Ocul Surf.

[33] Cui W., Chen S., Hu T., Zhou T., Qiu C., Jiang L. (2024). Nanoceria-mediated cyclosporin A delivery for dry eye disease management through modulating immune-epithelial crosstalk. ACS Nano.

[34] Wang Z., Koenig A.L., Lavine K.J., Apte R.S. (2019). Macrophage plasticity and function in the eye and heart. Trends Immunol.

[35] Doughty M.J., Jonuscheit S. (2019). Corneal structure, transparency, thickness and optical density (densitometry), especially as relevant to contact lens wear – a review. Contact Lens Anterior Eye.

[36] Ouyang W., Wu Y., Lin X., Wang S., Yang Y., Tang L. (2021). Tetrahedral framework nucleic acids ameliorate dry eye disease via inhibiting NLRP3 inflammasome activation. Invest Ophthalmol Vis Sci.

[37] Zhang X., Vimalin Jeyalatha M., Qu Y., He X., Ou S., Bu J. (2017). Dry eye management: targeting the ocular surface microenvironment. Int J Mol Sci.

[38] Willcox M.D.P., Argüeso P., Georgiev G.A., Holopainen J.M., Laurie G.W., Millar T.J. (2017). TFOS DEWS II tear film report. Ocul Surf.

[39] Dogru M., Kojima T., Simsek C., Tsubota K. (2018). Potential role of oxidative stress in ocular surface inflammation and dry eye disease. Invest Ophthalmol Vis Sci.

[40] Ji Y.W., Lee J.H., Choi E.Y., Kang H.G., Seo K.Y., Song J.S. (2020). Dry eye disease and corneal nerve alterations after refractive surgery. Invest Ophthalmol Vis Sci.

[41] Bae T., Hallis S.P., Kwak M.K. (2024). Hypoxia, oxidative stress, and the interplay of HIFs and NRF2 signaling in cancer. Exp Mol Med.

[42] Fuhrmann D.C., Brüne B. (2017). Mitochondrial composition and function under the control of hypoxia. Redox Biol.

[43] Willson J.A., Arienti S., Sadiku P., Reyes L., Coelho P., Morrison T. (2022). Neutrophil HIF-1α stabilization is augmented by mitochondrial ROS produced via the glycerol 3-phosphate shuttle. Blood.

[44] Wu K.K., Xu X., Wu M., Li X., Hoque M., Li G.H.Y. (2024). MDM2 induces pro-inflammatory and glycolytic responses in M1 macrophages by integrating iNOS-nitric oxide and HIF-1α pathways in mice. Nat Commun.

[45] Alam J., Yaman E., De Paiva C.S., Li D.Q., Villalba Silva G.C., Zuo Z. (2024). Changes in conjunctival mononuclear phagocytes and suppressive activity of regulatory macrophages in desiccation induced dry eye. Ocul Surf.

[46] Gui Q., Jiang Z., Zhang L. (2021). Insights into the modulatory role of cyclosporine A and its research advances in acute inflammation. Int Immunopharmacol.

