# Supplementary material for: Multi-crosslinked photocurable hydrogel for dry eye therapy: dual ROS scavenging and macrophage reprogramming for mitochondrial redox homeostasis and inflammation regulation through HIF-1/NF-κB suppression
Source: Asian J Pharm Sci. 2026 Oct 18;21(5):101209. doi: 10.1016/j.ajps.2026.101209 (PMC13760072; doi:10.1016/j.ajps.2026.101209)

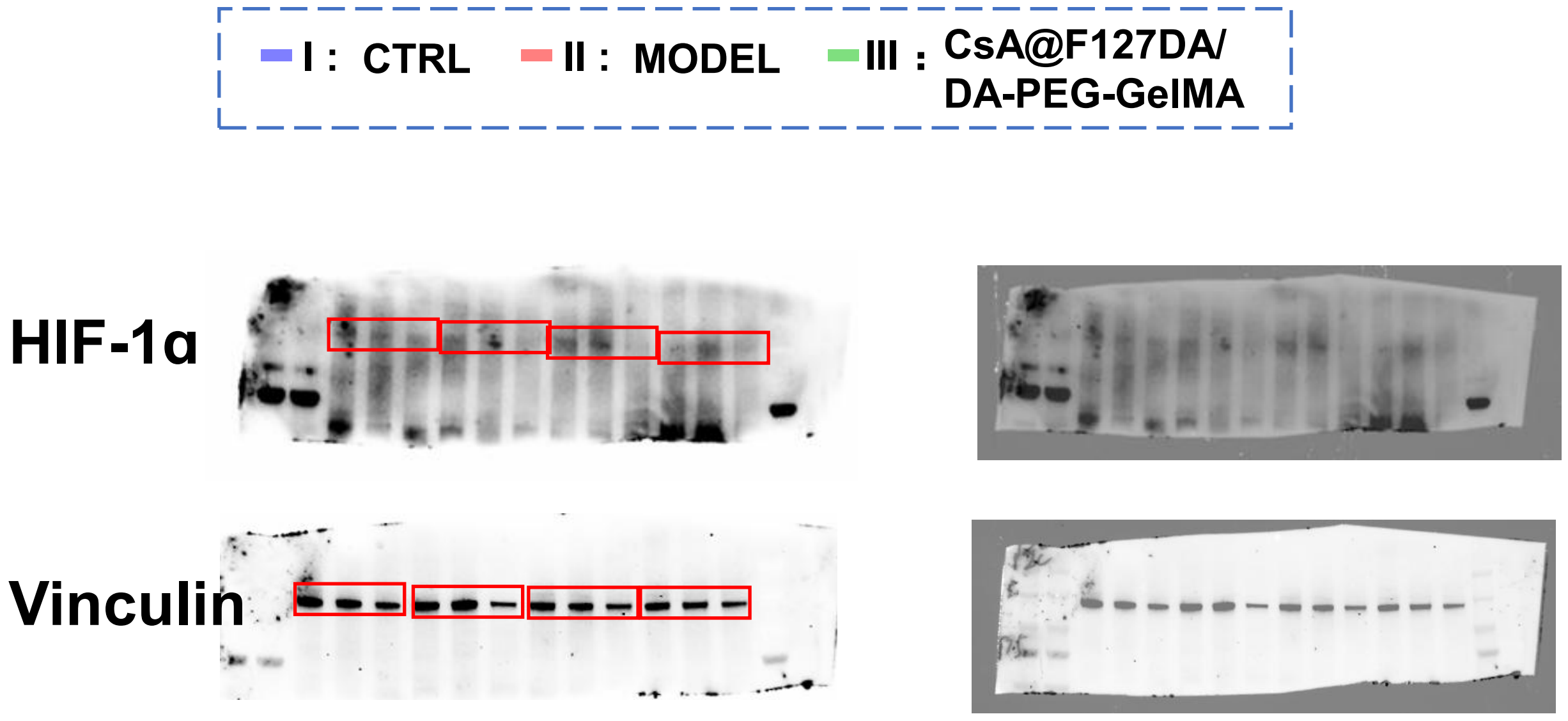
I : CTRL
II : MODEL
III : CsA@F127DA/ DA-PEG-GelMA
HIF-1α
Vinculin

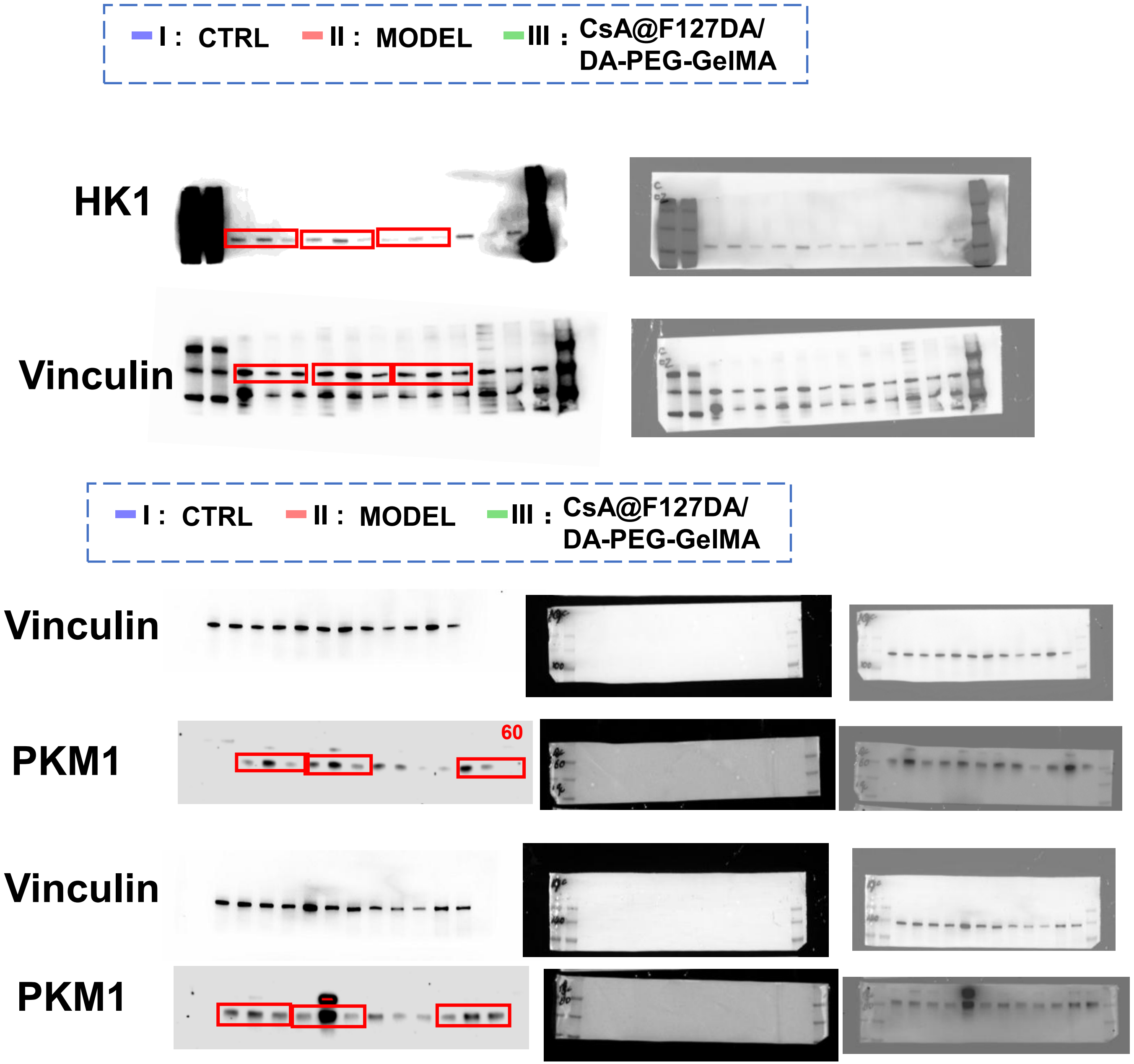
I： CTRL
II： MODEL
III： CsA@F127DA/ DA-PEG-GelMA
HK1
Vinculin
I： CTRL
II： MODEL
III： CsA@F127DA/ DA-PEG-GelMA
Vinculin
PKM1
60
Vinculin
PKM1

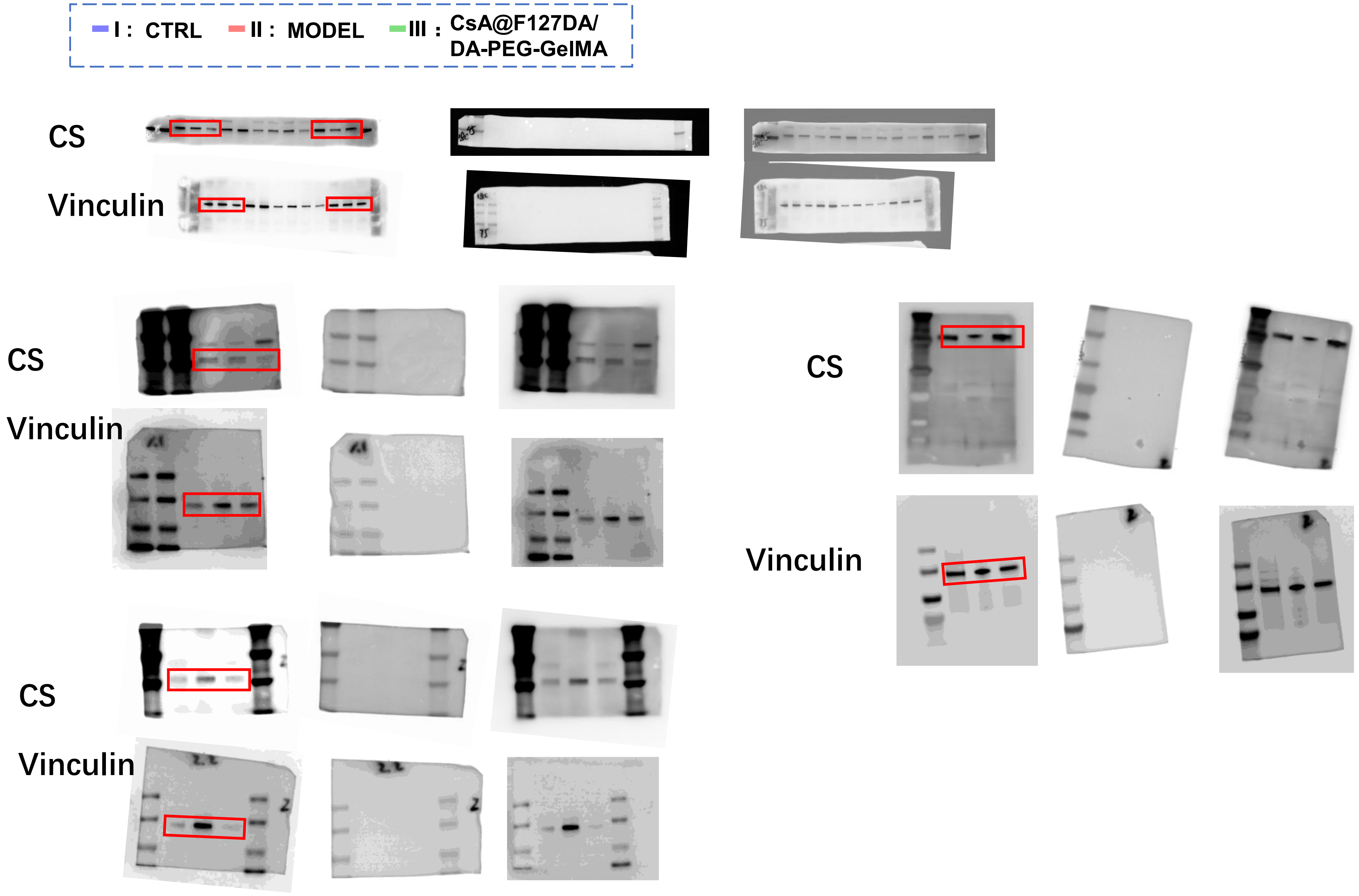
I： CTRL
II： MODEL
III： CsA@F127DA/ DA-PEG-GelMA
CS
Vinculin
CS
Vinculin
CS
Vinculin
CS
Vinculin

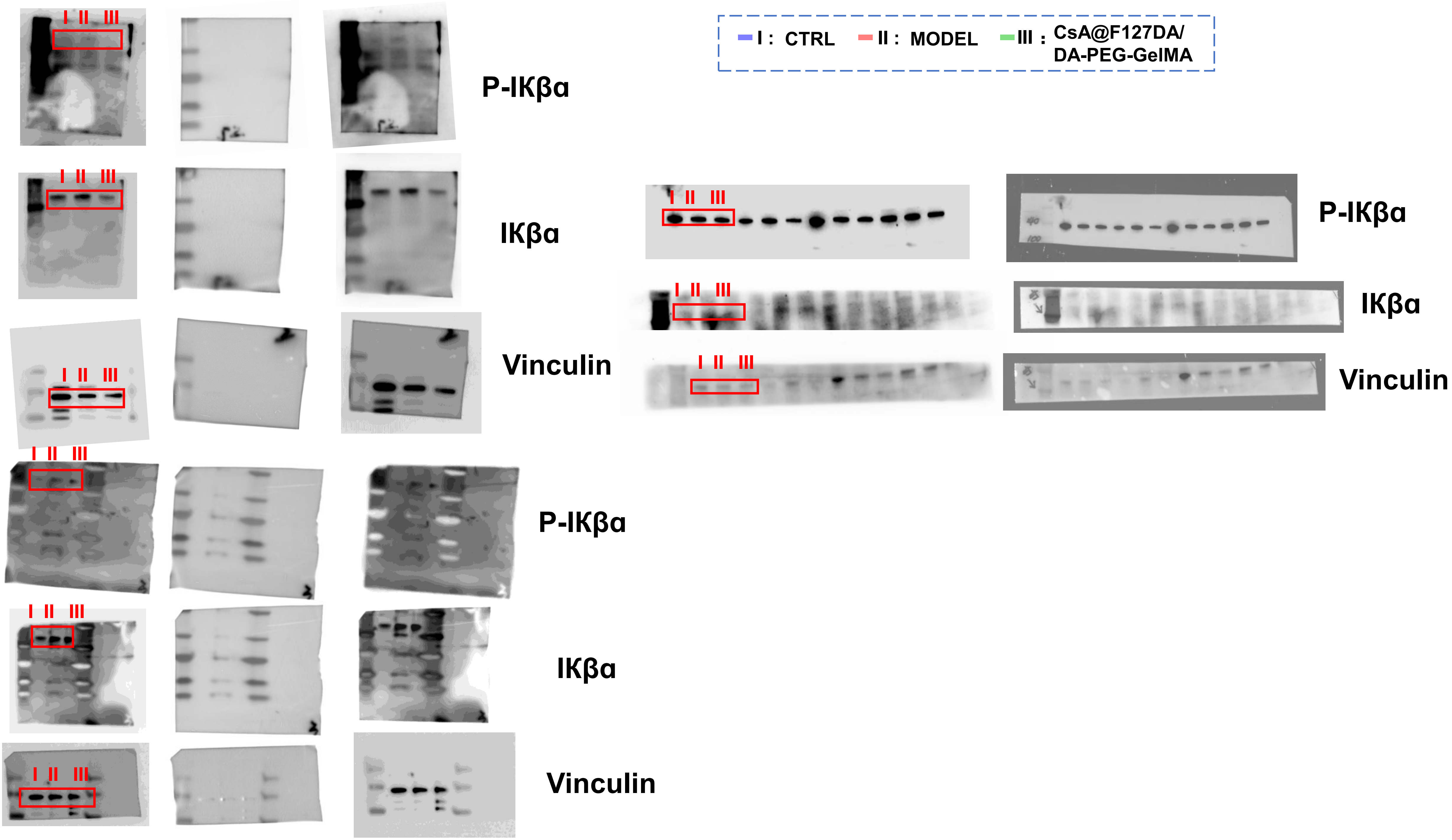
I : CTRL
II : MODEL
III : CsA@F127DA/DA-PEG-GelMA
P-IKβa
IKβa
Vinculin
P-IKβa
IKβa
Vinculin
P-IKβa
IKβa
Vinculin
I II III

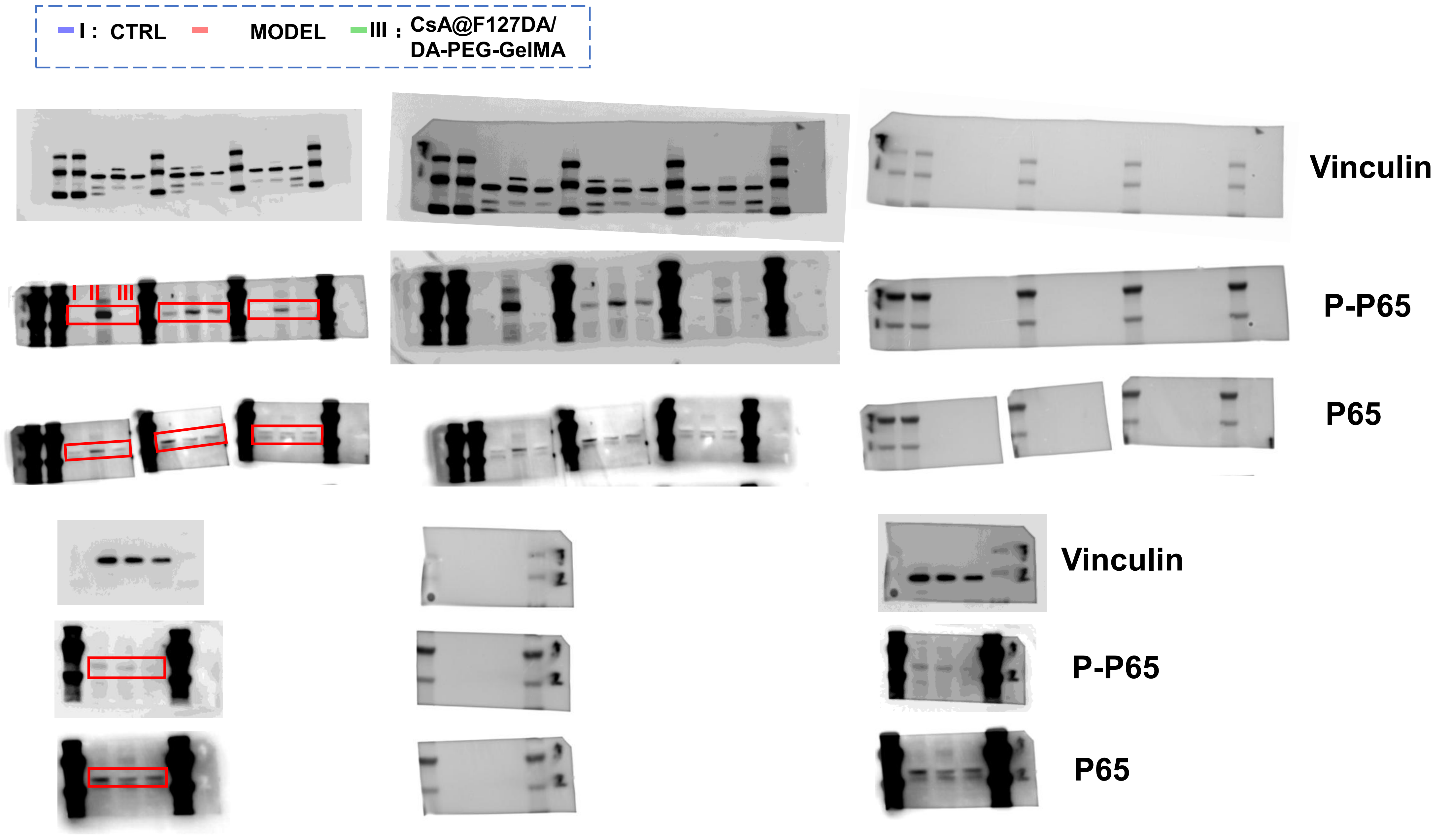
I : CTRL
MODEL
III : CsA@F127DA/ DA-PEG-GelMA
Vinculin
P-P65
P65
I II III
Vinculin
P-P65
P65

I: CTRL II: MODEL III : CsA@F127DA
IV :DA-PEG-GelMA V : CsA@F127DA/ DA-PEG-GelMA

Supplement

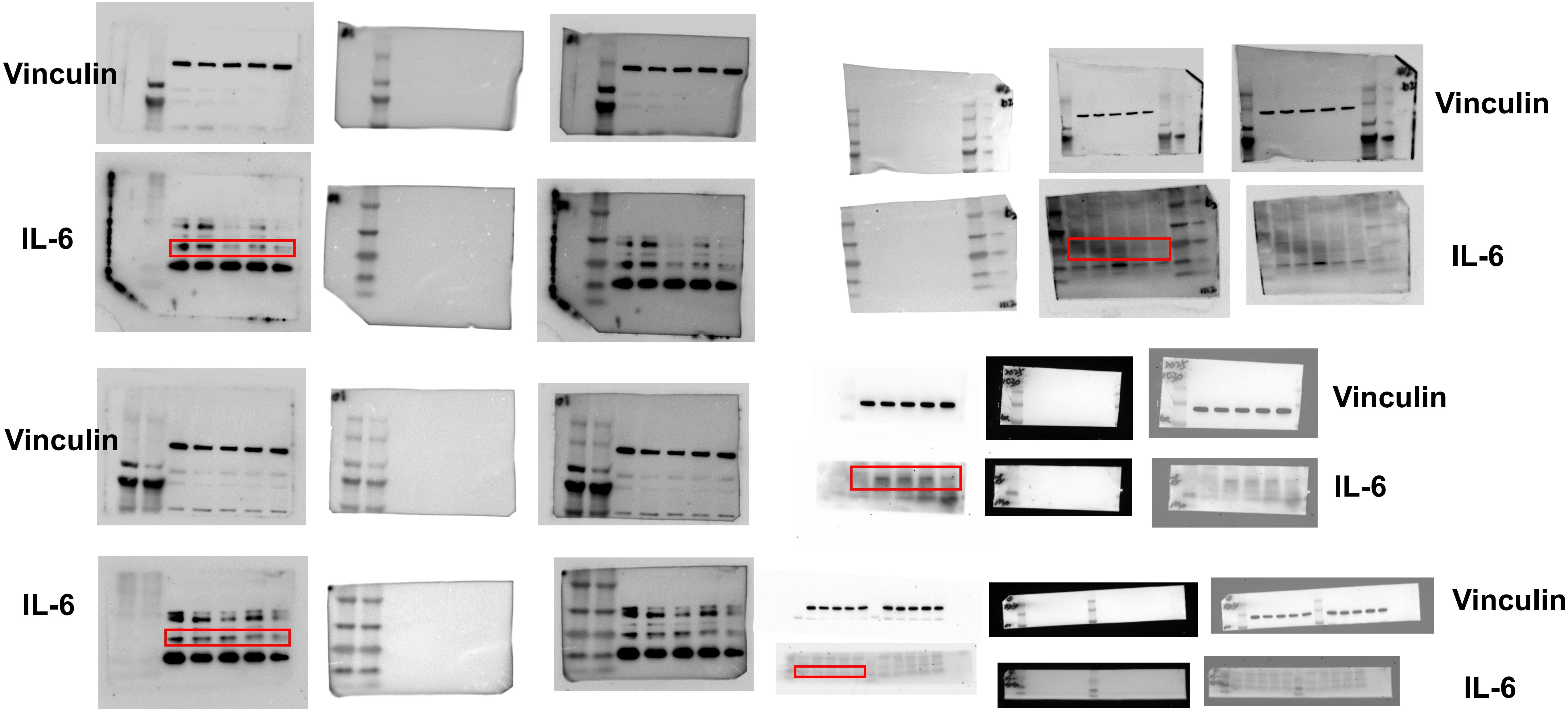

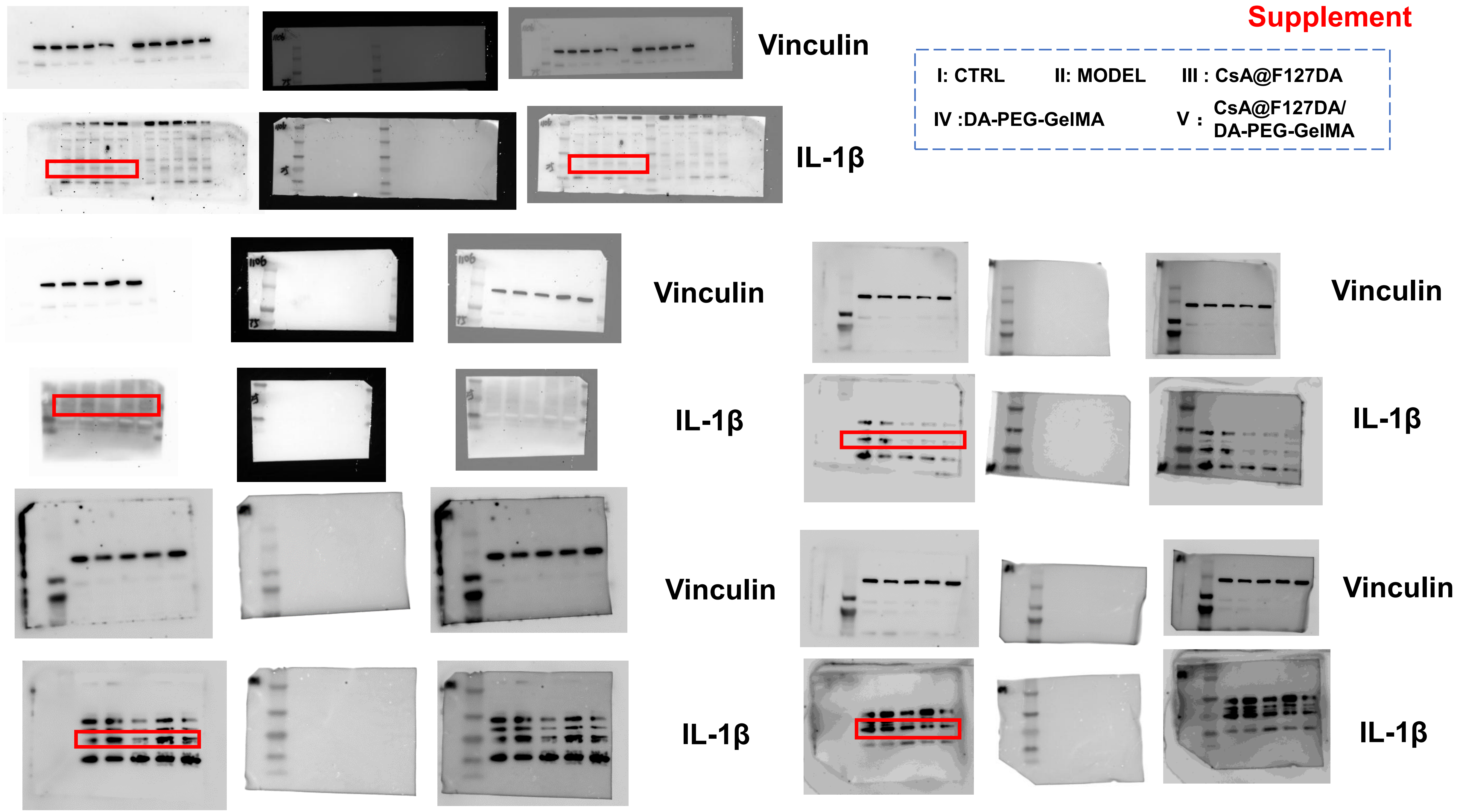

Supplement
I: CTRL
II: MODEL
III : CsA@F127DA
IV :DA-PEG-GelMA
V : CsA@F127DA/ DA-PEG-GelMA
Vinculin
IL-1β
Vinculin
IL-1β
Vinculin
IL-1β
Vinculin
IL-1β
Vinculin
IL-1β

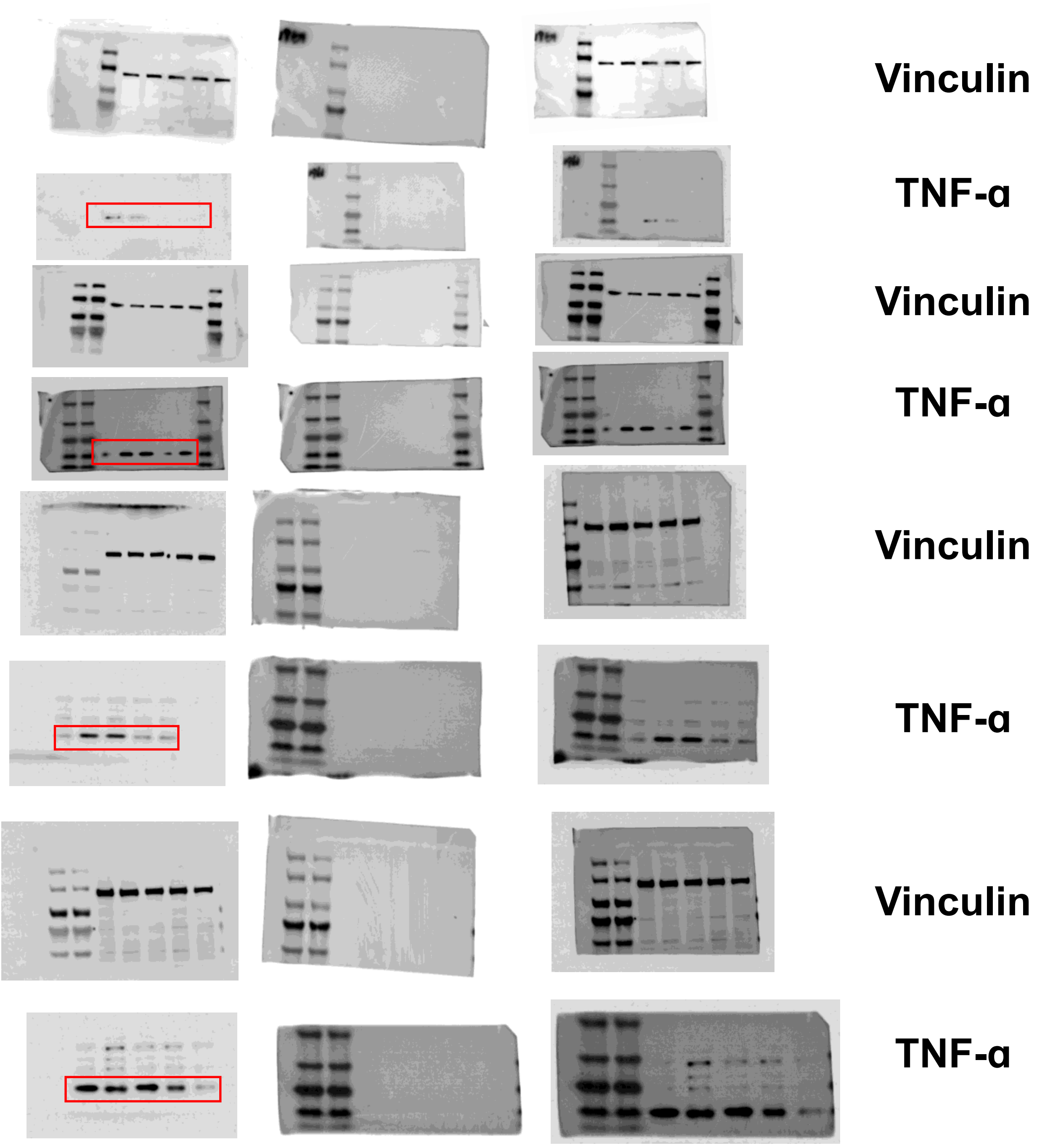

I: CTRL II: MODEL III : CsA@F127DA

IV :DA-PEG-GelMA V : CsA@F127DA/ DA-PEG-GelMA

Supplement

Supplement

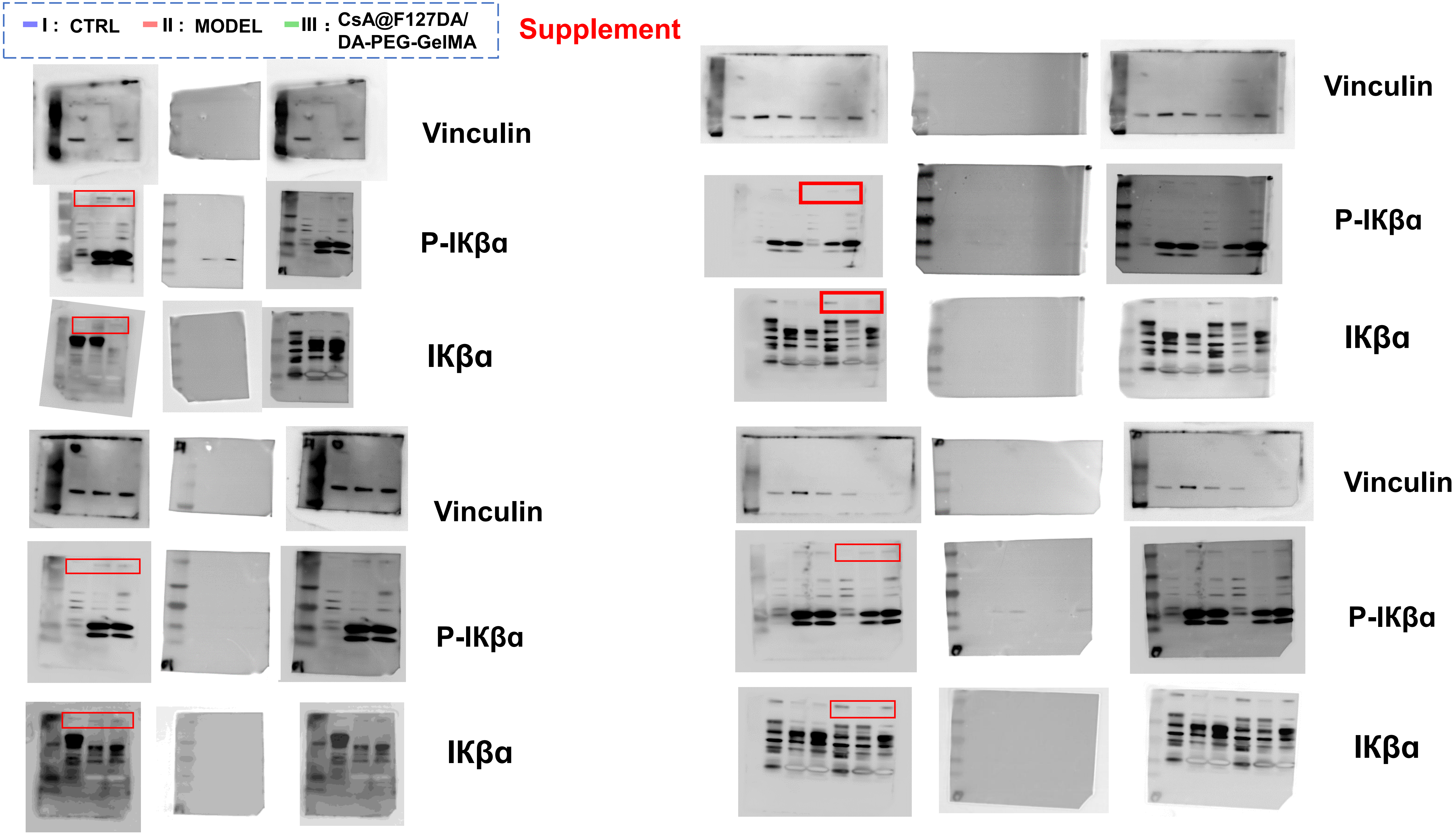

I : CTRL
II : MODEL
III : CsA@F127DA/ DA-PEG-GelMA
Vinculin
P-IKβa
IKβa
Vinculin
P-IKβa
IKβa
Vinculin
P-IKβa
IKβa
Vinculin
P-IKβa
IKβa

Supplement

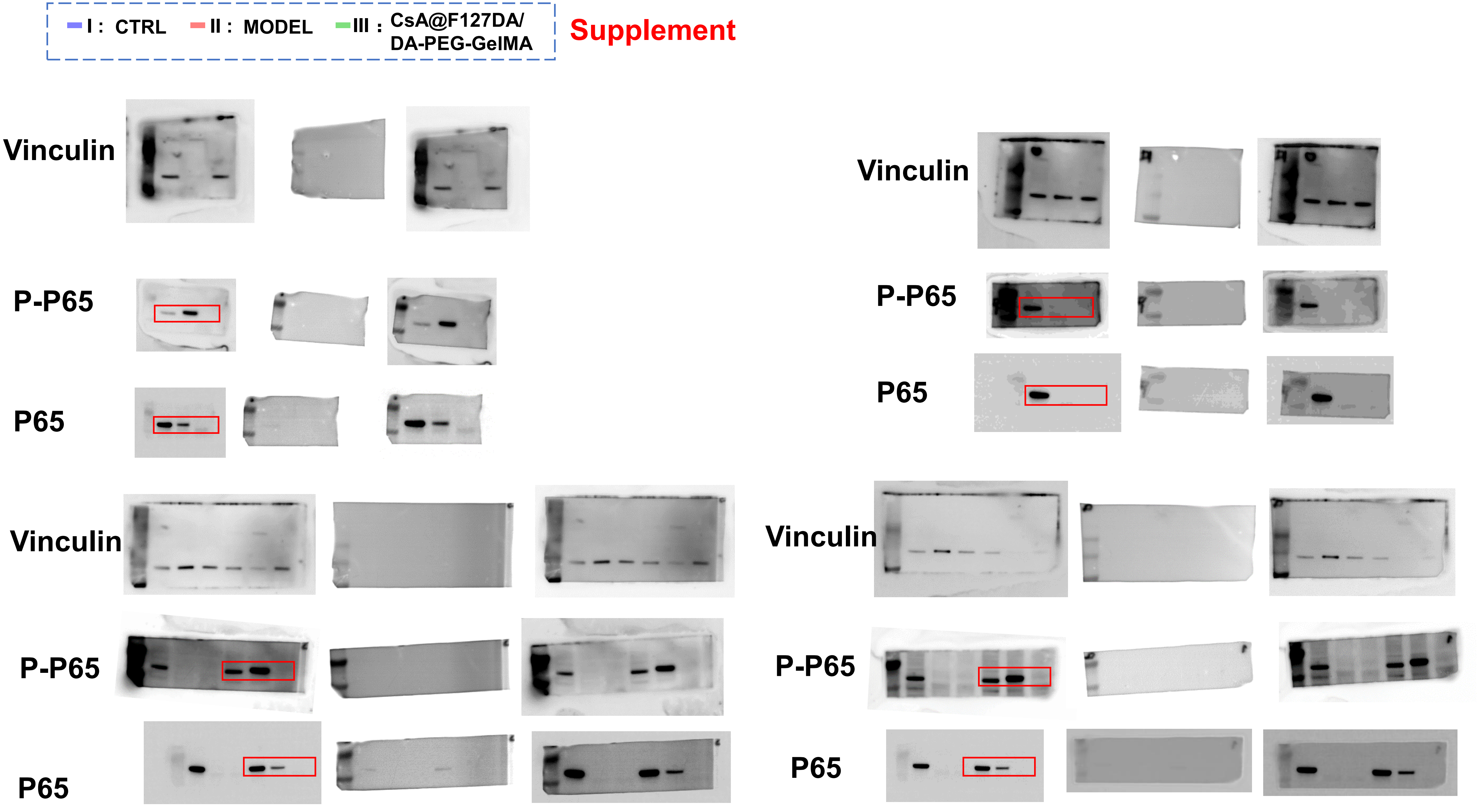

I : CTRL
II : MODEL
III : CsA@F127DA/ DA-PEG-GelMA
Vinculin
P-P65
P65
Vinculin
P-P65
P65
Vinculin
P-P65
P65
Vinculin
P-P65
P65

I : CTRL II : MODEL III : CsA@F127DA/DA-PEG-GelMA

Supplement

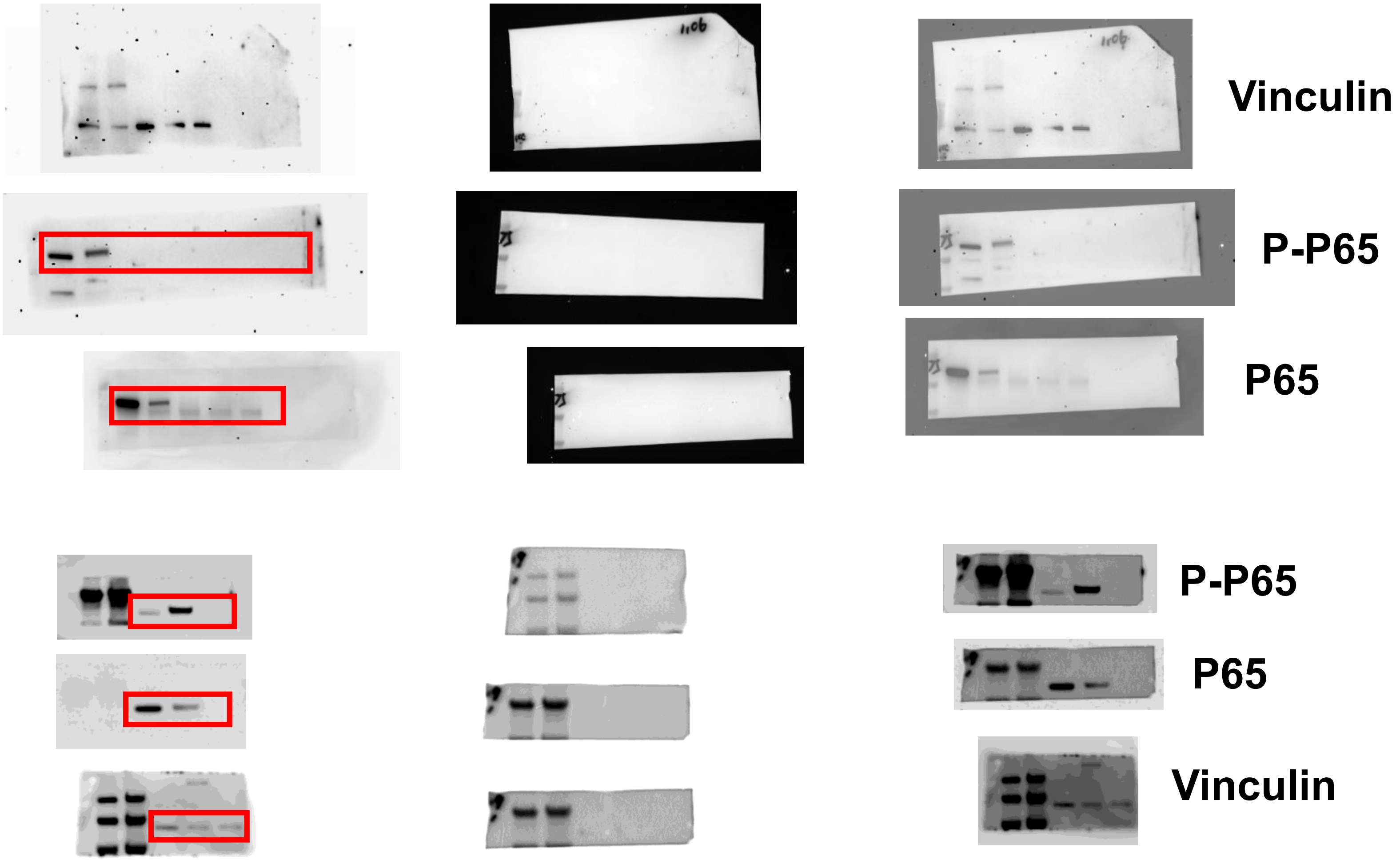

Supplement: Supplementary file 1 [file mmc5.pdf]
