# Supplementary material for: Multi-crosslinked photocurable hydrogel for dry eye therapy: dual ROS scavenging and macrophage reprogramming for mitochondrial redox homeostasis and inflammation regulation through HIF-1/NF-κB suppression
Source: Asian J Pharm Sci. 2026 Oct 18;21(5):101209. doi: 10.1016/j.ajps.2026.101209 (PMC13760072; doi:10.1016/j.ajps.2026.101209)
Supplement: Supplementary file 2 [file mmc1.docx]

**Supporting Information**

Qinghe Zhang^1,2#^, Ke Yan^1#^, Panqin Ma^1#^, Weilu He^1^, Minyan Wei^1^, Qiuping Liu^1*^, Yi Han^1*^, Zuguo Liu^2,1*^

1. Department of Ophthalmology, The First Affiliated Hospital of University of South China, Hengyang Medical School, University of South China, Hengyang Hunan 421001, China

2. Xiamen University affiliated Xiamen Eye Center, Fujian Provincial Key Laboratory of Ophthalmology and Visual Science, Fujian Engineering and Research Center of Eye Regenerative Medicine, Eye Institute of Xiamen University, School of Medicine, Xiamen University, Xiamen Fujian 361005, China

**^#^Co-first author means these authors contribute equally to this work**

***Corresponding author**

**Zuguo Liu, PhD, MD**

Department of Ophthalmology, The First Affiliated Hospital of University of South China, Hengyang Medical School, University of South China, Hengyang Hunan 421001, China

Email: zuguoliu@xmu.edu.cn

**Yi Han, PhD, MD**

Department of Ophthalmology, The First Affiliated Hospital of University of South China, Hengyang Medical School, University of South China, Hengyang Hunan 421001, China

Email: hanyixmu@foxmail.com; hanyi@usc.edu.cn

**Qiuping Liu, PhD, MD**

Department of Ophthalmology, The First Affiliated Hospital of University of South China, Hengyang Medical School, University of South China, Hengyang Hunan 421001, China

Email: 76655086@qq.com

**Figure S1. Characterization of F127DA and GelMA.** FT-IR spectra of GelMA, Gel, F127DA and F127.

**Figure S2. Characterization of AC-PEG-NHS, DA and DA-PEG-NHS.** (**A**) Nuclear magnetic resonance hydrogen spectroscopy (¹H NMR) of AC-PEG-NHS. (**B**) ¹H NMR of DA. (**C**) ¹H NMR of AC-PEG-NHS, DA, DA-PEG-AC.

**Figure S3.** **Time-module scanning of CsA@F127DA, DA-PEG-GelMA and CsA@F127DA/DA-PEG-GelMA.** (A) Time-modulus scanning of CsA@F127DA with strain and frequency characteristics. (B) Time-modulus scanning of DA-PEG-GelMA with strain and frequency characteristics. (C) Time-modulus scanning of CsA@F127DA/DA-PEG-GelMA with strain and frequency characteristics.

**Figure S4.** **The encapsulation rate of CsA and CsA loading capacity.** (A) The encapsulation rate of CsA in CsA@F127DA. (B) The CsA loading capacity in CsA@F127DA and CsA@F127DA/DA-PEG-GelMA. Data are expressed as mean ± standard error of the mean (S.E.M.). (n=4 per group).

**Figure S5.** **The concentration of DA and release ratio of DA.** (A) The concentration of DA in extract solution of DA-PEG-GelMA and CsA@F127DA/DA-PEG-GelMA. (B) The release ratio of DA in extract solution of DA-PEG-GelMA and CsA@F127DA/DA-PEG-GelMA.. Data are expressed as mean ± standard error of the mean (S.E.M.). (n=3 per group).

**Figure S6.** **Characterization of CsA@F127DA, DA-PEG-GelMA and CsA@F127DA/DA-PEG-GelMA.** Mass remained of hydrogels in collagenase I.

**Figure S7.** **Characterization of CsA@F127DA, DA-PEG-GelMA and CsA@F127DA/DA-PEG-GelMA.** (**A**) Grouping of hydrogels. (**B**) Swelling ratio of CsA@F127DA, DA-PEG-GelMA and CsA@F127DA/DA-PEG-GelMA. (**C**) Swelling rates of CsA@F127DA, DA-PEG-GelMA and CsA@F127DA/DA-PEG-GelMA. Data are expressed as mean ± standard error of the mean (S.E.M.). (n=3 per group). ^##^p < 0.05. ^#^ Points to a statistical comparison between this group and CsA@F127DA/DA-PEG-GelMA group.

**Figure S8.** **Characterization of CsA@F127DA, DA-PEG-GelMA and CsA@F127DA/DA-PEG-GelMA.** Water content of CsA@F127DA, DA-PEG-GelMA and CsA@F127DA/DA-PEG-GelMA. Data are expressed as mean ± standard error of the mean (S.E.M.). (n=3 per group). ^###^ p < 0.001. ^#^ Points to a statistical comparison between this group and CsA@F127DA/DA-PEG-GelMA group.

**Figure S9. Total antioxidant capacity of DA by ABTS Assay Kit.** The antioxidant performance of DA was assessed using the FRAP assay kit. Data are expressed as mean ± standard error of the mean (S.E.M.). (n=3 per group). ^***^ p < 0.001. ^*^Points to a statistical comparison between this group and Trolex group.

**Figure S10.** **CsA@F127DA/DA-PEG-GelMA presents good safety *in vitro*.** (**A**) Schematic illustration of hydrogel leachate production. (**B**) Relative cell viability of different hydrogel leachate on HCECs at 12h and 24h. Data are expressed as mean ± standard error of the mean (S.E.M.). (n=6 per group)

**Figure S11.** **CsA@F127DA/DA-PEG-GelMA presents good safety *in vitro*.** Representative live-dead cell staining image and percentage of living cells of different hydrogel leachate on HCECs at 24h. Data are expressed as mean ± standard error of the mean (S.E.M.). (n=3 per group).

**Figure S12.** **CsA@F127DA/DA-PEG-GelMA presents good safety *in vitro*.** Representative crystal violet staining image of different hydrogel leachate on HCECs at 24h.

**Figure S13.** **The biocompatibility of cells with different light exposure times.** (**A**) Relative cell viability under four 405 nm blue light exposure to irradiation for 10 s, 30 s, 60 s, and 180 s. Data are expressed as mean ± standard error of the mean (S.E.M.). (n=6 per group)

**Figure S14.** **Antioxidant properties of CsA@F127DA/DA-PEG-GelMA.** Representative images and fluorescence intensity quantification of SOD1 with different treatments under H_2_O_2_ oxidative stress. (n=6 per group). Data are expressed as mean ± standard error of the mean (S.E.M.). *p < 0.05, ***p < 0.001. ^#^p<0.05, ^##^p<0.01, ^###^p<0.001. * Points to a statistical comparison between this group and H_2_O_2_ group. ^#^ Points to a statistical comparison between this group and CsA@F127DA/DA-PEG-GelMA group.

**Figure S15.** **Antioxidant properties of CsA@F127DA/DA-PEG-GelMA.** Expression of SOD1 in HCECs with different treatments under H_2_O_2_ oxidative stress. (n=6 per group). Data are expressed as mean ± standard error of the mean (S.E.M.). *p < 0.05. ^#^p<0.05. * Points to a statistical comparison between this group and H_2_O_2_ group. ^#^ Points to a statistical comparison between this group and CsA@F127DA/DA-PEG-GelMA group.

**Figure S16.** **CsA@F127DA/DA-PEG-GelMA protects mitochondria.** Representative images and fluorescence intensity quantification of TOM20 staining with different hydrogel treatments under H_2_O_2_ oxidative stress. (n=4 per group). Data are expressed as mean ± standard error of the mean (S.E.M.). *p < 0.05. ^#^p<0.05. * Points to a statistical comparison between this group and H_2_O_2_ group. ^#^ Points to a statistical comparison between this group and CsA@F127DA/DA-PEG-GelMA group.

**Figure S17.** **CsA@F127DA/DA-PEG-GelMA protects mitochondria.** Quantification of ATP concentration with different hydrogel treatments under H_2_O_2_ oxidative stress. Data are expressed as mean ± standard error of the mean (S.E.M.). (n=4 per group). *p < 0.05. ^#^p<0.05, ^##^p<0.01. * Points to a statistical comparison between this group and H_2_O_2_ group. ^#^ Points to a statistical comparison between this group and CsA@F127DA/DA-PEG-GelMA group.

**Figure S18.** **CsA@F127DA/DA-PEG-GelMA promotes HCEs growth under HS stress.** Representative images of HCECs after scratch with different hydrogel treatments under HS oxidative stress. Data are expressed as mean ± standard error of the mean (S.E.M.). (n=5 per group). *p < 0.05, ***p < 0.001. ^#^p<0.05, ^##^p<0.01, ^###^p<0.001. * Points to a statistical comparison between this group and HS group. ^#^ Points to a statistical comparison between this group and CsA@F127DA/DA-PEG-GelMA group.

**Figure S19.** **CsA@F127DA/DA-PEG-GelMA protects mitochondria.** Representative images and fluorescence intensity quantification of TOM20 staining with different hydrogel treatments under HS oxidative stress. (n=6 per group). Data are expressed as mean ± standard error of the mean (S.E.M.). **p < 0.01. ^#^p<0.05, ^##^p<0.01. * Points to a statistical comparison between this group and HS group. ^#^ Points to a statistical comparison between this group and CsA@F127DA/DA-PEG-GelMA group.

**Figure S20.** **CsA@F127DA/DA-PEG-GelMA protects mitochondria.** Quantification of ATP concentration with different hydrogel treatments under HS oxidative stress. Data are expressed as mean ± standard error of the mean (S.E.M.). (n=4 per group). *p < 0.05, **p < 0.01, ***p < 0.001. ^#^p<0.05, ^##^p<0.01, ^###^p<0.001. * Points to a statistical comparison between this group and HS group. ^#^ Points to a statistical comparison between this group and CsA@F127DA/DA-PEG-GelMA group.

**Figure S21. Anti-inflammatory properties of CsA@F127DA/DA-PEG-GelMA.** Expression of IL-1β and IL-13 in HCECs treated with normal medium (CTRL), HS medium (HS), CsA@F127DA，DA-PEG-GelMA，CsA@F127DA/DA-PEG-GelMA (n=5 to 6 per group). Data are expressed as mean ± standard error of the mean (S.E.M.). *p<0.05, ***p<0.001. ^#^p<0.05, ^##^p<0.01, ^###^p<0.001. * Points to a statistical comparison between this group and HS group. ^#^ Points to a statistical comparison between this group and CsA@F127DA/DA-PEG-GelMA group.

**Figure S22. Anti-inflammatory properties of CsA@F127DA/DA-PEG-GelMA. (A)** Grouping of HCECs treated with normal medium (CTRL), HS medium (HS), CsA@F127DA，DA-PEG-GelMA，CsA@F127DA/DA-PEG-GelMA. **(B)** Protein expression of IL-6 and IL-1β in HCEs treated with normal medium (CTRL), HS medium (HS), CsA@F127DA，DA-PEG-GelMA, CsA@F127DA/DA-PEG-GelMA. **(C)** Statistical analysis of protein expression level of IL-6 (left) and IL-1β (right) in HCEs treated with normal medium (CTRL), HS medium (HS), CsA@F127DA，DA-PEG-GelMA，CsA@F127DA/DA-PEG-GelMA (n = 5 per group). Data are expressed as mean ± standard error of the mean (S.E.M.). *p<0.05. ^#^p<0.05. * Points to a statistical comparison between this group and HS group. ^#^ Points to a statistical comparison between this group and CsA@F127DA/DA-PEG-GelMA group.

**Figure S23. Anti-inflammatory properties of CsA@F127DA/DA-PEG-GelMA.** Expression of TNF-α in HCECs treated with normal medium (CTRL), H_2_O_2_ medium (H_2_O_2_), CsA@F127DA，DA-PEG-GelMA, CsA@F127DA/DA-PEG-GelMA (n=4 to 6 per group). Data are expressed as mean ± standard error of the mean (S.E.M.). *p<0.05, ***p<0.001. ^#^p<0.05, ^##^p<0.01, ^###^p<0.001. * Points to a statistical comparison between this group and H_2_O_2_ group. ^#^ Points to a statistical comparison between this group and CsA@F127DA/DA-PEG-GelMA group.

**Figure S24. Anti-inflammatory properties of CsA@F127DA/DA-PEG-GelMA. (A)** Grouping of HCECs treated with normal medium (CTRL), H_2_O_2_ medium (H_2_O_2_), CsA@F127DA, DA-PEG-GelMA, CsA@F127DA/DA-PEG-GelMA. **(B)** Protein expression of TNF-α in HCEs treated with normal medium (CTRL), H_2_O_2_ medium (H_2_O_2_), CsA@F127DA, DA-PEG-GelMA, CsA@F127DA/DA-PEG-GelMA. **(C)** Statistical analysis of protein expression level of TNF-α in HCEs treated with normal medium (CTRL), H_2_O_2_ medium (H_2_O_2_), CsA@F127DA, DA-PEG-GelMA, CsA@F127DA/DA-PEG-GelMA (n = 4 per group). Data are expressed as mean ± standard error of the mean (S.E.M.). *p<0.05, **p<0.01. ^##^p<0.01. * Points to a statistical comparison between this group and H_2_O_2_ group. ^#^ Points to a statistical comparison between this group and CsA@F127DA/DA-PEG-GelMA group.

**Figure S25. Safety of CsA@F127DA/DA-PEG-GelMA *in vivo*.** Schematic illustration for safety experiments.

**Figure S26. Safety of CsA@F127DA/DA-PEG-GelMA *in vivo*.** Representative images of corneal white and fluorescent sodium staining by slit-lamp.

**Figure S27. Safety of CsA@F127DA/DA-PEG-GelMA *in vivo*.** Representative images of *in vivo* corneal laser confocal microscope images.

**Figure S28. Safety of CsA@F127DA/DA-PEG-GelMA *in vivo*.** (**A**)Representative OCT images of anterior segment. (**B**) Representative OCT images of posterior segment. (**C**) Corneal and retinal thickness statistics (n=3 to 4 per group). Data are expressed as mean ± standard error of the mean (S.E.M.).

**Figure S29. Safety of CsA@F127DA/DA-PEG-GelMA *in vivo*.** Representative images of fundus photography and fundus fluorescein imaging.

**Figure S30. Safety of CsA@F127DA/DA-PEG-GelMA *in vivo*.** Representative images of H&E staining of corneal sections, conjunctiva sections, optic nerve sections and retina sections.

**Figure S31. Safety of 405nm blue light exposure for 3 months. Representative images of the corneal white part captured by slit lamp and fluorescent sodium staining.**

**Figure S32. Safety of 405nm blue light exposure for 3 months.** Representative images of *in vivo* corneal laser confocal microscope images.

**Figure S33. Safety of 405nm blue light exposure for 3 months.** Representative OCT images of anterior segment.

**Figure S34. Safety of 405nm blue light exposure for 3 months.** Representative images of fundus photography and fundus fluorescein imaging.

**Figure S35. Safety of 405nm blue light exposure for 3 months.** (A) Representative images of H&E staining of anterior segment, including corneal sections, conjunctiva sections. (B) Representative images of H&E staining of posterior segment,including optic nerve sections and retina sections.

**Figure S36. Safety of 405nm blue light exposure for 3 months.** Representative images of H&E staining of the main organs - heart, liver and kidney.

**Figure S37. Quantitative comparison of corneal thickness among different groups.** Corneal thickness was measured using OCT imaging (A) and H&E-stained sections (B). Data are expressed as mean ± Standard Error of the Mean (S.E.M.). *p<0.05, **p<0.01, ***p<0.001. * Points to a statistical comparison between this group and MODEL group.

**Figure S38.** **CsA@F127DA/DA-PEG-GelMA maintains corneal redox balance**. Expression of SOD2 in Cornea with different treatments under dry eye. Data are expressed as mean ± standard error of the mean (S.E.M.) (n=6 per group). ***p<0.001. ^#^p<0.05, ^##^p<0.01, ^###^p<0.001.* Points to a statistical comparison between this group and MODEL group. ^#^ Points to a statistical comparison between this group and CsA@F127DA/DA-PEG-GelMA group.

**Figure S39.** **CsA@F127DA/DA-PEG-GelMA improves corneal inflammation and apoptosis.** (**A**) Gene set enrichment analysis (GSEA) diagram illustrating the enrichment of inflammatory response in model group and CsA@F127DA/DA-PEG-GelMA group. (**B**) Gene set enrichment analysis (GSEA) diagram illustrating the enrichment of apoptosis in model group and CsA@F127DA/DA-PEG-GelMA group.

**Figure S40.** **CsA@F127DA/DA-PEG-GelMA improves corneal barrier function.** Representative images and positive cell count of corneal TUNEL staining under dry eye (the red arrow indicates the area of corneal epithelium TUNEL staining) (n=3 to 4 per group). Data are expressed as mean ± standard error of the mean (S.E.M.).

**Figure S41.** **CsA@F127DA/DA-PEG-GelMA maintains the immune homeostasis of the conjunctiva** (**A**) Representative images of conjunctival CD86 under DE with different hydrogels effects. (**B**) Representative images of conjunctival CD206 under DE with different hydrogels effects.

**Figure S42.** **CsA@F127DA/DA-PEG-GelMA inhibites conjunctival inflammation.** Gene set enrichment analysis (GSEA) diagram illustrating the enrichment of IL-6 pathway (**A**), integrin pathway (**B**) in model group and CsA@F127DA/DA-PEG-GelMA group.

**Figure S43.** **CsA@F127DA/DA-PEG-GelMA inhibites conjunctival inflammation and apoptosis.** Gene set enrichment analysis (GSEA) diagram illustrating the enrichment of apoptosis (**A**) and cell adhesion (**B**) in model group and CsA@F127DA/DA-PEG-GelMA group.

**Figure S44.** **CsA@F127DA/DA-PEG-GelMA improves conjunctival oxidative phosphorylation.** Gene set enrichment analysis (GSEA) diagram illustrating the enrichment of oxidative phosphorylation in model group and CsA@F127DA/DA-PEG-GelMA group.

**Figure S45. CsA@F127DA/DA-PEG-GelMA downregulates the NF-κB pathway in M1 polarized RAW264.7 cells.** (A) Grouping of RAW264.7 cells treated with normal medium (CTRL), M1 polarization medium (MODEL), CsA@F127DA/DA-PEG-GelMA. (B) The protein level of, P-P65, P65, P-Iκβα, and Iκβα in RAW 264.7 treated with normal medium (CTRL), M1 polarization medium (MODEL), CsA@F127DA/DA-PEG-GelMA. (C) Statistical analysis of the protein expression of P-P65 (n = 6 per group) (left) and P-Iκβα (n = 4 per group) (right) in RAW 264.7 treated with normal medium (CTRL), M1 polarization medium (MODEL), CsA@F127DA/DA-PEG-GelMA. Data are expressed as mean ± Standard Error of the Mean (S.E.M.). *p<0.05. #p<0.05. * Points to a statistical comparison between this group and MODEL group. # Points to a statistical comparison between this group and CsA@F127DA/DA-PEG-GelMA group.

**Figure S46**. **CsA@F127DA/DA-PEG-GelMA improves meibomian gland** **atrophy.** Macroscopic image of the meibomian glands under different treatments. The red triangle indicates the meibomian glands defect.

**Figure S47**. **CsA@F127DA/DA-PEG-GelMA improves meibomian gland atrophy.** H&E staining of meibomian glands under different treatments.

**Figure S48**. **CsA@F127DA/DA-PEG-GelMA improves lipid secretion of meibomian gland.** Oil-red staining of meibomian glands under different treatments.

**Figure S49**. **CsA@F127DA/DA-PEG-GelMA improves lipid secretion of meibomian gland.** LipidTOX immunofluorescence staining of meibomian glands under different treatments.
