# Supplementary material for: Multi-crosslinked photocurable hydrogel for dry eye therapy: dual ROS scavenging and macrophage reprogramming for mitochondrial redox homeostasis and inflammation regulation through HIF-1/NF-κB suppression
Source: Asian J Pharm Sci. 2026 Oct 18;21(5):101209. doi: 10.1016/j.ajps.2026.101209 (PMC13760072; doi:10.1016/j.ajps.2026.101209)
Supplement: Supplementary file 3 [file mmc2.docx]

**Caption 1.** Protein expression of HIF-1α, HK1, PKM1, CS and Vinculin in cornea under different treatments. The left side presents protein bands without marker and the right side presents protein bands with marker. The red boxes circled are the bands presented in the article.

**Caption 2.** Protein expression of P-Iκβα, Iκβα, P65, P-P65 and Vinculin in conjunctiva under different treatments. The left side presents protein bands without marker and the right side presents protein bands with marker. The red boxes circled are the bands presented in the article.

**Caption 3.** The protein expression of IL-6, IL-1β and Vinculin in conjunctiva under different treatments. The left side presents protein bands without marker and the right side presents protein bands with marker. The red boxes circled are the bands presented in the article.

**Caption 4.** The protein expression of TNF-ɑ and Vinculin in conjunctiva under different treatments. The left side presents protein bands without marker and the right side presents protein bands with marker. The red boxes circled are the bands presented in the article.

**Caption 5.** The protein expression of P-P65, P65, P-Iκβα, Iκβα, and Vinculin in conjunctiva under different treatments. The left side presents protein bands without marker and the right side presents protein bands with marker. The red boxes circled are the bands presented in the article.
